# Burden of hereditary angioedema: results from a multinational survey of caregivers for adult and pediatric patients

**DOI:** 10.1186/s13023-025-04123-2

**Published:** 2026-02-12

**Authors:** Maureen Watt, Inmaculada Martinez-Saguer, Angela Simon, Ryan Murphy, Marie De La Cruz, Ricardo Zwiener, Mauricio Sarrazola, Anete S. Grumach

**Affiliations:** 1 Takeda Development Center Americas, Inc., Lexington, MA USA; 2 HZRM Hemophilia Center Rhine Main, Frankfurt/Main, Germany; 3 ICON, Raleigh, NC USA; 4 Servicio de Alergia e Inmunología Clínica, Hospital Universitario Austral, Pilar, Buenos Aires, Argentina; 5 Departamento de Medicina, Grupo GIPPAM, Universidad de Pamplona, Cúcuta, Colombia; 6 Clinical Immunology, Faculdade de Medicina, Centro Universitario Faculdade de Medicina ABC (CEUFMABC), Santo Andre, Brazil

**Keywords:** Hereditary angioedema, Survey, Noninterventional, Burden of disease, Caregiver, Quality of life

## Abstract

**Background:**

Hereditary angioedema (HAE), a rare autosomal dominant disorder characterized by recurrent, potentially life-threatening attacks of cutaneous or submucosal swelling, affects patients’ everyday activities and psychological well-being. Although caregivers are instrumental in helping patients cope with HAE, its impact on the caregivers’ quality of life is poorly documented. Using web-based surveys (July 2022‒February 2023), this international study (Argentina, Brazil, Colombia, Croatia, Denmark, Germany, Hungary, Ireland, Norway, Poland, Portugal, Romania, and Sweden) assessed the humanistic and psychosocial burden of caregivers (≥ 18 years old) of pediatric (< 18 years) and adult (≥ 18 years) patients with diagnosed HAE.

**Results:**

In total, 120 caregivers completed the surveys: 54 caregivers of pediatric patients (CoPs; mean age 40.6 years; 79.6% female) and 66 caregivers of adult patients (CoAs; mean age 42.7 years; 48.5% female). CoPs and CoAs reported 5.6 and 13.1 HAE attacks (mean) in the past 6 months for individuals receiving their care, respectively. CoPs provided care for 23.5 days (mean) per month on average; in the past 4 weeks, CoPs missed (mean) 2.6 days (mean) of work, while the children missed 3.9 days (mean) of school. CoPs cited a lack of understanding of their caregiving duties from schools (20.4%), employers/coworkers (16.7%), family (13.0%), friends (13.0%), and partner/spouse (13.0%). CoPs reported impacts on their work (37.0%), sleep (37.0%), and household chores (31.5%), and restricted time with family (29.6%), spouses/partners (27.8%), and friends (24.1%). Emotional impacts on the CoPs included worry about the child’s health (90.7%) and future (68.5%); CoPs themselves reported having sleep problems (24.1%), migraine (22.2%), gastrointestinal disorders (22.2%), and anxiety (20.4%). CoAs reported impacts on their work (28.8%), sleep (28.8%), and recreational activities (27.3%), leading to missing time of work (mean 0.94 days in past 4 weeks). Emotional impacts on the CoAs included worry about the health of individual they provide care for (92.4%) and future (40.9%); CoAs themselves reported having anxiety (13.6%), migraines (13.6%), and sleep problems (12.1%).

**Conclusion:**

Results of this caregiver survey revealed that the caregiver role in HAE is time-demanding and adversely impacts various aspects of the caregiver’s life, particularly their emotional wellbeing.

**Supplementary Information:**

The online version contains supplementary material available at 10.1186/s13023-025-04123-2.

## Introduction

Hereditary angioedema (HAE) is a rare autosomal dominant genetic disorder characterized by unpredictable, painful, recurrent, and potentially life-threatening attacks of cutaneous or submucosal swelling [1–3]. The disease is caused either by a deficiency of the C1 inhibitor (C1INH; HAE-C1INH-Type1) or by the production of dysfunctional C1INH (HAE-C1INH-Type2) [1]; a third, rarer type of HAE (HAE-nC1INH) is associated with normal C1INH levels and activity [4]. Globally, the prevalence of diagnosed HAE in the general population is estimated to range between 1 in 50,000 to 1 in 100,000 people [5, 6].

Disease symptoms typically present before 20 years of age (mean age at onset is estimated as 11 years) and persist throughout the patients’ lifetime [2, 7]. HAE most commonly affects the extremities, gastrointestinal tract, and face [2]. HAE attacks can also affect the larynx, which can potentially be fatal due to asphyxiation [2]. Abdominal attacks, caused by gastrointestinal edema, can be debilitating, manifesting as severe abdominal spasms, while laryngeal edema is the primary cause of mortality in HAE [8]. Attack frequency is unpredictable, ranging from multiple episodes per week to less than 1 per year, with attack rates being lower in children than in adults [9]. Untreated HAE attacks typically last for several days before resolving spontaneously [9, 10]. Disease management includes on-demand treatment of acute attacks and long-term prophylaxis (LTP) to minimize the frequency and severity of future HAE attacks [11, 12].

Studies of the burden of illness in HAE, conducted predominantly in western Europe, Scandinavia, and North America, have highlighted the detrimental impact of HAE on patients’ activities of daily living, work productivity, and psychological well-being, with many reporting depression and anxiety, fear of future attacks, sleep disturbances, and guilt about absence from work, school, and social obligations [3, 13–19]. The unpredictability of HAE attacks and, in many instances, the absence of obvious triggers, adds to the burden of the disease on patients’ physical and psychological well-being [20]. Disease management guidelines emphasize that a key role in HAE treatment is to improve the patients’ quality of life by recognizing and then ameliorating those factors that contribute to the burden of disease in HAE [11, 12]. The economic burden of HAE on patients is reflected through direct costs associated with management of the disease (medication, medical visits, supportive care) and indirect costs such as loss of work productivity, school productivity/attendance, hindered career or educational advancement, and income [21–23].

Many patients with HAE have an informal caregiver, often a family member, who may also have HAE [24]. Caregivers are instrumental in helping patients manage everyday challenges, including navigating access to care, providing support in a wide range of activities, influencing patient decisions, and assisting with treatment [24]. The quality-of-life burden experienced by caregivers is less well documented in publications than that experienced by patients with HAE. Improved understanding of the impact of HAE on caregivers’ lives would help to inform disease management, as would information on the disease burden experienced by those patients outside western Europe and North America. To this end, an international survey was undertaken to assess the humanistic and psychosocial burden on caregivers of individuals with HAE, and to gather information on disease burden among patients themselves in these countries.

## Methods

### Study design and population

This was a noninterventional, cross-sectional, web-based study of caregivers of pediatric and adult patients with HAE conducted in Europe (Croatia, Denmark, Germany, Hungary, Ireland, Norway, Poland, Portugal, Romania, and Sweden) and South America (Argentina, Brazil, and Colombia) between July 2022 and February 2023. Two separate surveys were conducted for caregivers of children and caregivers of adults; a third survey was conducted in adults with HAE. The three surveys were developed simultaneously by Takeda and translated into the official language of each country. Pilot testing was conducted ahead of the surveys to assess participants’ interpretation of survey instructions and content, and the consistency of their responses. The protocol was registered at ISRCTN registry (ISRCTN85479564).

Institutional Review Board or Ethics Committee approval was obtained before recruitment and data collection for each site or country (both central and local ethics committee approval were required in Brazil, Croatia and Germany). This study was conducted in accordance with the protocol, the Declaration of Helsinki, Good Pharmacoepidemiology Practices, International Society of Pharmacoepidemiology guidelines for Good Pharmacoepidemiology Practices, and local regulations. All participants provided informed consent before recruitment and data collection.

Caregivers of pediatric patients were recruited by local HAE International-affiliated patient advocacy organizations (Croatia, Denmark, Hungary, Norway, Poland, Romania, and Sweden) or via local healthcare providers (Argentina, Brazil, Colombia, Germany, Ireland, and Portugal). Recruitment materials were developed for the telephone/e-mail invitation, website advertisement, and social media postings and distributed by recruitment partners. Caregivers of adult patients were recruited directly through referral by patients with HAE who were completing a complementary, simultaneous, multinational patient survey (an adaptation of the version described by Mendivil et al. [13]) to assess the impact of HAE on quality of life and work productivity. Adult patients with HAE were asked if an adult caregiver provided regular care or support on an unpaid, voluntary basis because of their HAE. Patients with HAE who answered “yes” received a unique link to the caregiver survey, for the patient to email to their caregiver. The surveys were administered from July 3, 2022, through February 8, 2023.

For study inclusion, caregivers were required to be aged ≥ 18 years; to self-identify as being a primary caregiver providing regular care or support on an unpaid voluntary basis to an adult (*≥* 18 years) or pediatric (< 18 years) patient diagnosed with HAE (HAE-C1INH-Type1; HAE-C1INH-Type2; HAE-C1INH undifferentiated [caregiver is unsure of exact type of HAE, but it is either HAE-C1INH-Type1 or HAE-C1INH-Type2]; HAE-nC1INH; or unknown [caregiver does not know what type of HAE the patient has]); to be fluent in the local language; and to provide their informed consent. On completion of the survey, participants were provided with a unique completion code to send to the local recruiting partner. Where local laws and regulations allowed monetary remuneration (Denmark, Germany, Hungary, Norway, Romania and Sweden), payment was offered to caregivers for completion of the survey for their first dependent (30 USD or local equivalent) and any subsequent dependent (15 USD); in Germany, a retail voucher for 25 EUR was issued. Local ethical guidelines and regulations in Argentina, Brazil, Colombia, Croatia, Ireland, Poland, and Portugal did not allow participant remuneration.

### Study assessments

Surveys were administered through an online portal (Confirmit) and were self-reported. The surveys included items covering both the patient and the caregiver on demographic characteristics, clinical and disease characteristics, economic burden of HAE, and humanistic burden of HAE. The pediatric caregiver survey included the following sections: Patient information; Patient’s most recent angioedema attack; Impact of HAE on the child/adolescent patient; Caregiving information; Caregiver impacts; and Background and sociodemographic information on the caregiver. The adult caregiver survey included the following sections: Patient information; Caregiving information; Impacts on the caregiver; and Background and sociodemographic information on the caregiver. Survey data were analyzed for the total participant sample and for predefined subgroups, including country and patient age group (for the survey of caregivers of pediatric patients; <12 years and 12 to < 18 years). All of the patient-reported outcome data in adult patients were directly elicited (self-assessed) in the adult patient survey; the patient-reported outcome measures were not completed by caregivers on behalf of their adult patients. The effect of recurrent angioedema on patients’ health-related quality of life over the past 4 weeks in the adult patient survey was assessed using the Angioedema Quality of Life Questionnaire (AE-QoL), which covers the Functioning, Fatigue/Mood, Fears/Shame, and Nutrition domains [25]. AE-QoL total score is transformed to a linear scale ranging from 0 to 100, with a score of 100 indicating maximum possible impairment of health-related quality of life. Patients’ perception of disease control over the last 3 months in the adult patient survey was assessed using the self-administered Angioedema Control Test (AECT) questionnaire, which addresses the frequency of angioedema, angioedema-related quality-of-life impairment, the unpredictability of angioedema attacks, and angioedema control achieved with current treatment [26]. The impact of recurrent angioedema on patients’ ability to engage in paid and unpaid work and other social activities during the past 7 days in the adult patient survey was assessed using the Work Productivity and Activity Impairment: Global Health questionnaire (WPAI: GH), which covers absenteeism (percent work time missed), presenteeism (percent reduced productivity while working), overall work productivity loss (an estimate combining absenteeism and presenteeism), and activity impairment (percent impairment in daily non-work-related activities) [27]. The general health over the past week in the adult patient survey was assessed using the 12-item Short Form Health Survey (SF-12 v2), which has 12 questions in eight domains and two summary scores: Physical Component Summary and Mental Component Summary [28, 29].

Study findings were summarized with descriptive statistics: mean ± standard deviation (SD) for continuous variables, and n (%) for categorical variables.

## Results

### Survey of caregivers of pediatric patients

#### Study population

For the pediatric caregiver survey, 54 caregivers of 55 pediatric patients with HAE were recruited from 10 countries (Argentina, Brazil, Colombia, Croatia, Denmark, Germany, Hungary, Ireland, Poland, and Portugal): one caregiver was responsible for two pediatric patients.

Self-reported characteristics of caregivers of pediatric patients are summarized in Table 1. The mean age of caregivers of pediatric patients was 40.6 years and approximately 80% were female; almost one-half (46.3%) had HAE themselves. Demographic and clinical characteristics of the pediatric patients under their care are reported in Table 2 (data by country in Additional file 2: Supplementary Table 1): the pediatric patients were of mean age 10.6 years, and approximately one-half were aged < 12 years. A breakdown of the patients’ demographic and clinical characteristics by age group is provided in Additional file 2: Supplementary Table 2. The majority of pediatric patients had HAE-C1INH ; overall, the mean age at diagnosis of HAE was 4.2 (range, 0.0‒14.0) years.

**Table 1:** Characteristics of caregivers of pediatric patients with HAE

Caregiver characteristic	Caregivers *N* = 54^a^
Age, years	
Mean ± SD	40.6 ± 8.3
Range	23.0–55.0
Sex, n (%)	
Female	43 (79.6)
Male	11 (20.4)
Employment status, n (%)	
Employed full time	21 (38.9)
Employed part time	10 (18.5)
Self-employed	10 (18.5)
Stay-at-home parent/homemaker	12 (22.2)
Other	1 (1.8)

All data presented in this table are self-reported by survey participants

*HAE* hereditary angioedema, *SD* standard deviation

^a^One caregiver participated for two pediatric patients and therefore was not asked the questions of demographics or impacts multiple times

**Table 2:** Caregiver-reported demographic and clinical characteristics of pediatric patients with HAE

Patient characteristic	Pediatric patients *N* = 55
Age, years	
Mean ± SD	10.6 ± 3.8
Median (IQR)	11.0 (8.0–14.0)
Range	1.0–17.0
Age range, n (%)	
< 12 years	30 (54.5)
≥ 12 years	25 (45.5)
Sex, n (%)	
Female	28 (50.9)
Male	27 (49.1)
Type of HAE, n (%)	
HAE-C1INH^a^	52 (94.5)
HAE-nC1INH	2 (3.6)
Unknown^b^	1 (1.8)
Age at HAE onset, years	
Mean ± SD	4.5 ± 3.9
Median (IQR)	4.0 (2.0–6.0)
Age at HAE diagnosis, years	
Mean ± SD	4.2 ± 3.6
Median (IQR)	4.0 (1.0–6.0)

All data presented in this table are self-reported by survey participants

*HAE* hereditary angioedema, *IQR* interquartile range, *HAE-C1INH* hereditary angioedema due to C1 inhibitor deficiency, *HAE-nC1INH* hereditary angioedema due to normal C1 inhibitor, *SD* standard deviation

^a^Includes patients for whom the caregiver selected the answer “HAE Type I”, “HAE Type II”, or “Unsure of exact HAE type, but it is either HAE Type I or II” to the survey question “Which type of HAE does the patient have?”

^b^Includes patients for whom the caregiver selected the answer “I don’t know what type of HAE” to the survey question “Which type of HAE does the patient have?”

#### Disease burden on pediatric patients

Among the pediatric patients, caregivers reported 5.6 ± 20.0 (mean ± SD) HAE attacks in the past 6 months, with 45.5% of patients experiencing their most recent attack within the previous 4 weeks, and 18.2% within the past 7 days. Caregivers reported having prescription for on-demand treatment to treat HAE attacks for most but not all of the children under their care (43/55, 78.2%). Caregivers also reported prescriptions for medications for long-term prophylaxis (LTP) in nearly half (23/55, 41.8%) of the children under their care. On-demand medications reported by the caregivers mainly included icatibant (35/43 pediatric patients using on-demand treatment, 81.4%) and C1INH (7/43 pediatric patients using on-demand treatment, 16.3%). Caregivers reported that 12/23 (52.2%) pediatric patients currently on LTP were using LTP for < 1 year, 6/23 (26.1%) were using LTP for a duration between 1 year to < 3 years, and 5/23 (21.7%) were using LTP for ≥ 3 years. LTP medications reported by the caregivers included HAE-specific medications indicated for on-demand treatment only (8/23, 34.8%), lanadelumab (1/23, 4.3%), C1INH (1/23, 4.3%), danazol (1/23, 4.3%), and “other” medications (4/23, 17.4%).

**Fig. 1:**
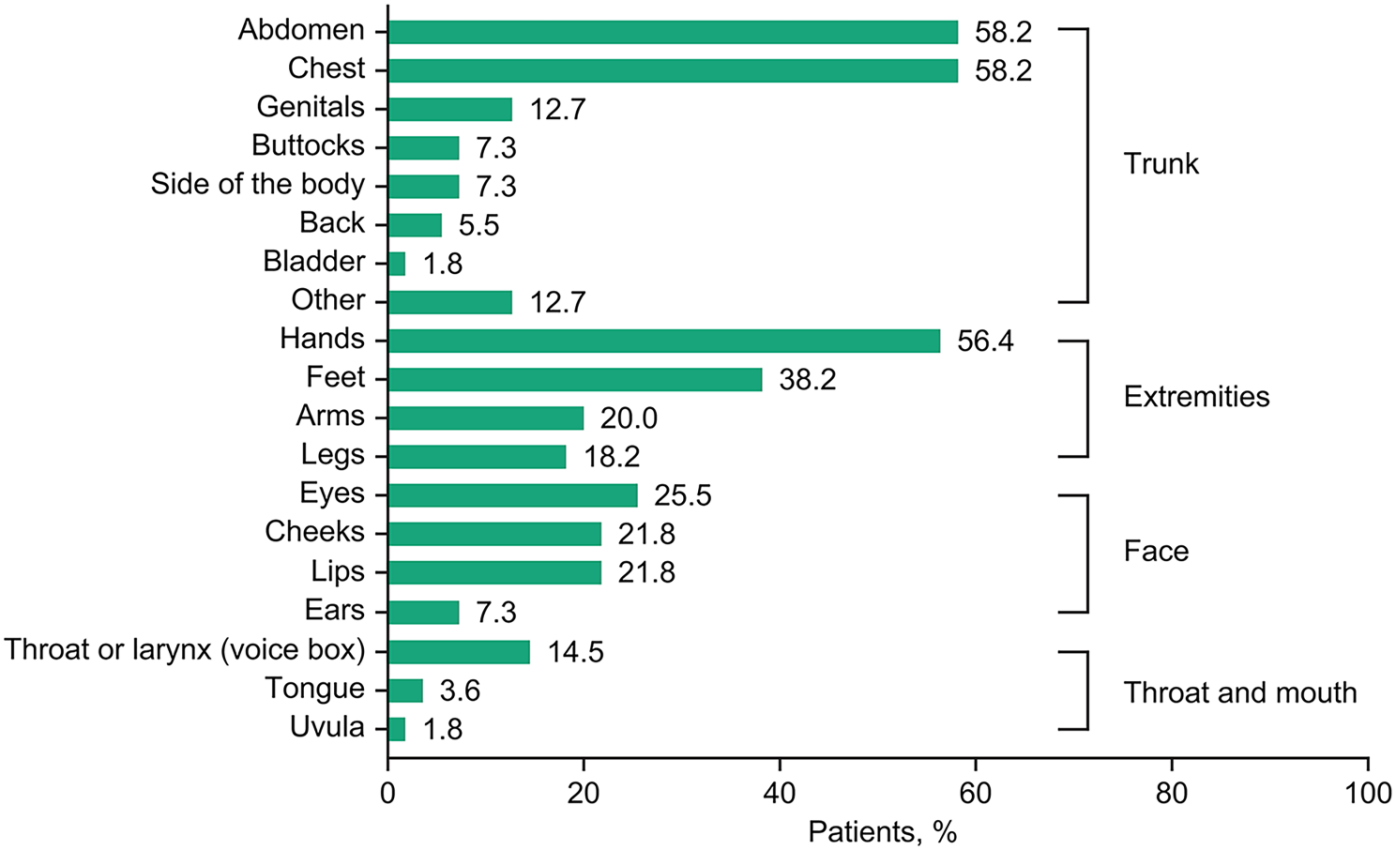
Areas of the body affected by any HAE attack ever experienced in pediatric patients with HAE. The sum of categories may exceed 100%, as responses were not mutually exclusive. *HAE* hereditary angioedema

In the pediatric patients, body areas affected by any HAE attack ever experienced included the abdomen, chest, hands, and voice box/larynx (Fig. 1). A country-by-country breakdown of the most frequently affected body sites is provided in Additional file 2: Supplementary Table 3.

#### Quality-of-life burden on caregivers of pediatric patients

In the 4 weeks before completing the survey, caregivers of pediatric patients reported being absent from work for 2.6 ± 5.8 days (mean ± SD; median, 1.0 day), while the patients in their care missed 3.9 ± 6.2 days of school (mean ± SD; median, 2.0 days) over the same period. Caregivers reported providing care for 23.5 ± 11.8 (mean ± SD) days per month.

**Fig. 2:**
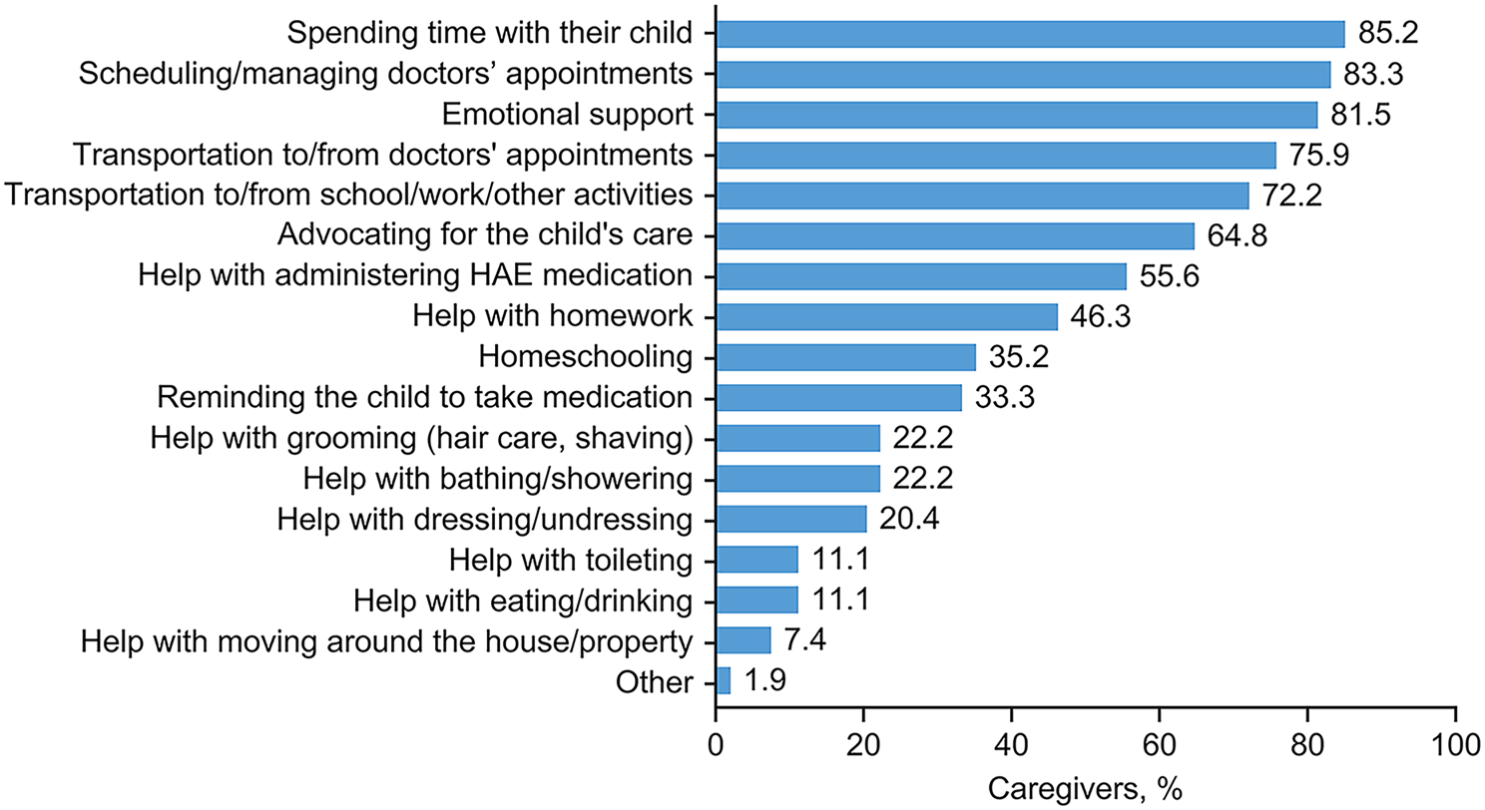
Type of care provided by caregivers of pediatric patients with HAE. *HAE* hereditary angioedema

Caregivers reported a lack of understanding among family and colleagues of the time they spent in providing care to pediatric patients; many reported a lack of understanding from the child’s school (20.4%), from their employer/coworkers (16.7%), family (13.0%), friends (13.0%), and partner/spouse (13.0%). The most common types of help that they felt they offered were spending time with their child, scheduling/managing doctors’ appointments, and providing emotional support (over 80% of participants for each). A full breakdown of the types of help provided by caregivers of pediatric patients is shown in Fig. 2 (a breakdown of data by country is provided in Additional file 2: Supplementary Table 4).

**Fig. 3:**
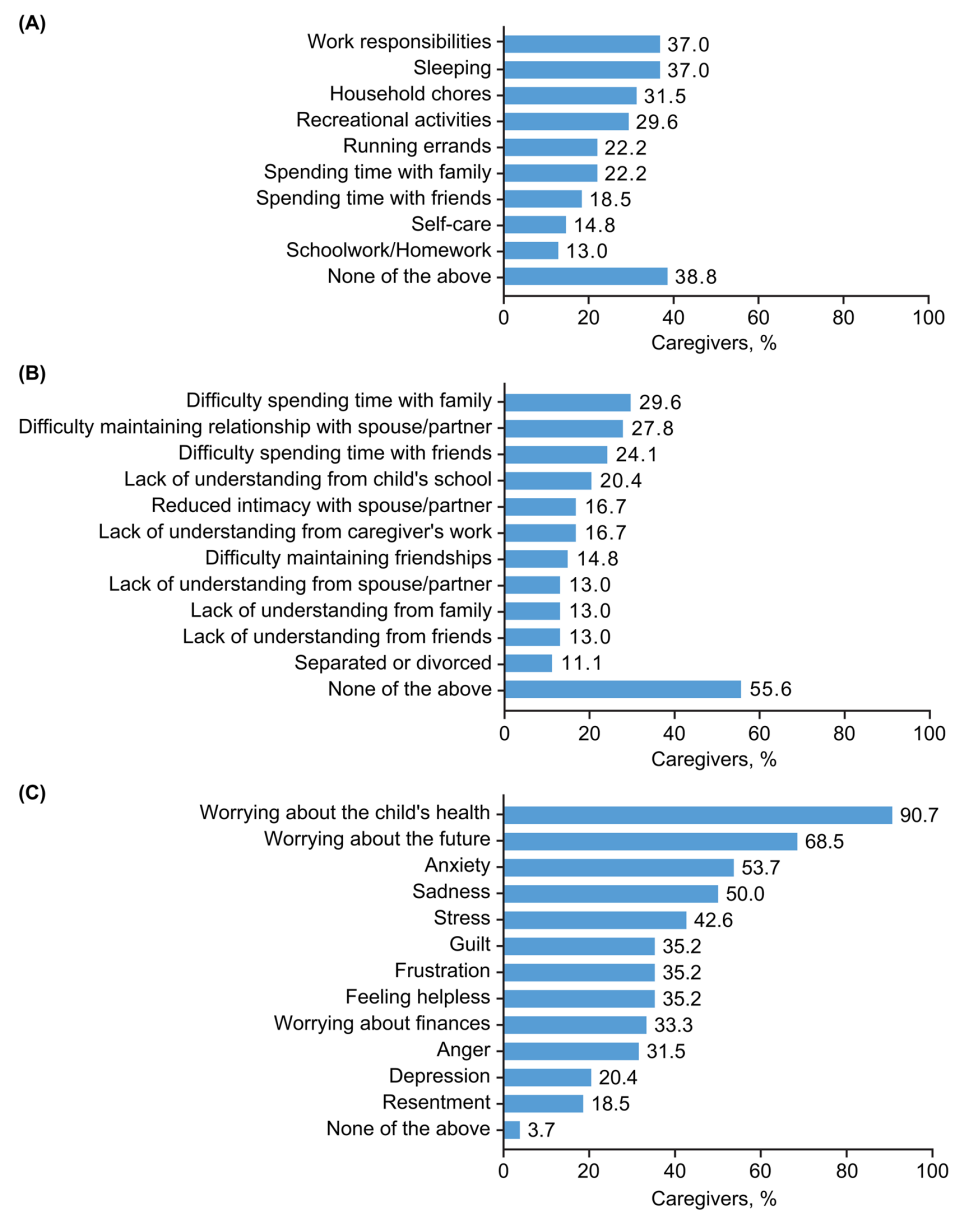
Self-reported impact on (**A**) activities (**B**) relationships and (**C**) emotional well-being of caregiving to pediatric patients with HAE. *HAE* hereditary angioedema

Caregivers of pediatric patients reported that their caregiving responsibilities limited their ability to plan their own lives in different ways, notably, to work full time (33.3%), to advance in their professional careers (25.9%), to pursue their personal interests or hobbies (25.9%), their freedom to travel or go on holiday (24.1%), and to relocate to another city or country (9.3%). Caregivers reported that providing pediatric care particularly impacted their work responsibilities, sleep, and household chores (over 30% of participants for each); other activities affected are depicted in Fig. 3A (data by country is provided in Additional file 2: Supplementary Table 5). Caregivers also reported difficulties spending time with their family and friends, and difficulties maintaining their relationships with spouses/partners (approximately 25–30% of participants for each), with divorce/separation being cited as a direct or indirect consequence by over one-tenth of caregivers (Fig. 3B; data by country is provided in Additional file 2: Supplementary Table 6). In addition, caregivers identified a number of serious emotional impacts connected with their caregiving responsibilities, as depicted in Fig. 3C (country-specific data are presented in Additional file 2: Supplementary Table 7). The majority reported that caring for a pediatric patient was associated with worries about the child’s health and the future.

A large proportion of caregivers of pediatric patients reported having their own health conditions and symptoms (74.1%), including sleep problems (24.1%), migraine (22.2%), gastrointestinal disorders/symptoms (22.2%), and anxiety (20.4%). In the last 4 weeks, 35.2% of caregivers of pediatric patients with HAE reported that they felt sadness due to the patient’s HAE some, most, or all of the time; 48.1% reported spending some, most, or all of the time feeling stress or worry about the patient’s HAE, and 35.2% reported having “not at all” or “a little” time to themselves while caring for a pediatric patient with HAE.

### Survey of caregivers of adult patients

#### Study population

Of the 260 adult patients who completed the complementary patient survey, 120 (46.2%) reported having a caregiver and were provided with the caregiver survey link to forward to their caregiver. In total, 66 caregivers from nine countries (Argentina, Brazil, Colombia, Denmark, Germany, Hungary, Ireland, Portugal, Sweden) completed the adult caregiver survey. Self-reported characteristics of caregivers of adult patients with HAE are summarized in Table 3.

Caregivers of adult patients had a mean age of 42.7 years, and approximately half were female. The majority (75.8%) lived in the same household as the patient, approximately 50% were employed full time, and slightly over one-half were the spouse/partner of the patient. Overall, caregivers of adult patients had been providing care for a mean ± SD 15.0 ± 10.5 years and were currently providing care for 14.4 ± 12.4 days per month. Demographic and clinical characteristics of the adult patients (*n* = 66) receiving support from these caregivers are reported in Table 4 (data by country in Additional file 2: Supplementary Table 8). These patients had a mean age of 40.1 years, and the majority had HAE-C1INH; caregivers reported some form of LTP in most patients. Nearly one-half (42.4%) of caregivers of adult patients reported that they usually received additional support from a hired professional; however, only one-quarter (25.8%) had ever requested access to support from social services or other resources, with 10.6% accessing these forms of support in the past 4 weeks.

**Table 3:** Characteristics of caregivers of adult patients with HAE

Caregiver characteristic	Caregivers *N* = 66
Age, years	
Mean ± SD	42.7 ± 12.8
Range	21.0–71.0
Sex, n (%)	
Female	32 (48.5)
Male	34 (51.5)
Employment status, n (%)	
Employed full time	32 (48.5)
Self-employed	14 (21.2)
Employed part time	8 (12.1)
Retired	4 (6.1)
Stay-at-home parent/homemaker	3 (4.5)
Student	1 (1.5)
On sick or extended leave	1 (1.5)
Furloughed or temporarily out of work	1 (1.5)
Other	2 (3.0)
Relationship to patient with HAE, n (%)	
Spouse/partner	36 (54.5)
Daughter or son	14 (21.2)
Mother or father	10 (15.2)
Other family member	4 (6.1)
Mother-in-law or father-in-law	1 (1.5)
Other	1 (1.5)
Living in the same household as patient with HAE, n (%)	50 (75.8)
People in the household with HAE	
Mean ± SD	1.8 ± 0.9
Range	1.0–4.0

All data presented in this table are self-reported by survey participants

*HAE* hereditary angioedema, *SD* standard deviation

**Table 4:** Demographic and clinical characteristics of adult patients with HAE as reported by their caregivers

Patient characteristic	Adult patients *N* = 66
Age, years	
Mean ± SD	40.1 ± 14.4
Range	18.0–81.0
Sex, n (%)	
Female	42 (63.6)
Male	24 (36.4)
Type of HAE, n (%)	
HAE-C1INH^a^	63 (95.5)
Unknown^b^	3 (4.5)
Current LTP use, n (%)	
Yes	40 (60.6)
No	25 (37.9)
Unknown	1 (1.5)

All data presented in this table are self-reported by survey participants

*HAE* hereditary angioedema, *LTP* long-term prophylaxis, *SD* standard deviation

^a^Includes patients for whom the caregiver selected the answer “HAE Type I”, “HAE Type II”, or “Unsure of exact HAE type, but it is either HAE Type I or II” to the survey question “Which type of HAE does the patient have?”

^b^Includes patients for whom the caregiver selected the answer “I don’t know what type of HAE” to the survey question “Which type of HAE does the patient have?”

#### Disease burden on adult patients

Based on the available dataset and coding information, it was possible to pair 47 of the 66 caregivers of adults with their own adult patients who completed the patient survey; typographical errors or errors in data capture may have prevented matching the remaining caregivers with their patients. In this cohort, the caregivers reported 13.1 ± 16.5 (mean ± SD) HAE attacks in the previous 6 months, compared with 14.0 ± 15.9 (mean ± SD) attacks reported by the patients themselves. Patients rated their most recent HAE attack as severe/very severe (14/46 with available data; 30.4%), moderate (23/46 with available data, 50.0%) or mild/very mild (9/46 with available data, 19.6%), with a mean duration of 3.2 (range 1.0‒6.0) hours. Information on LTP use (available for 28 of the 47 patients) indicated that fewer than 30% of patients currently receiving LTP were using one of the first-line LTP medications recommended by current guidelines (C1INH, lanadelumab, or berotralstat).

Directly elicited HRQoL outcome data from the cohort of 47 adult patients with HAE yielded a mean ± SD AE-QoL score of 42.8 ± 25.4, with approximately one-half of patients displaying moderate to large impairment in HRQoL as indicated by an AE-QoL total score ≥ 39 [30]. In the last 3 months, patients recorded an overall mean ± SD AECT score of 6.9 ± 3.2, and approximately three-quarters of patients (74.5%) had AECT score of < 10, indicating patient perception of poorly controlled disease [31]. Impairments in patients’ work and activity during the past 7 days due to their HAE were reflected in their WPAI: GH questionnaire scores, which indicated an average loss of 13.4% of work time (absenteeism; data available for 35 patients), average reduction of 28.8% in workplace performance (presenteeism; data available for 32 patients), 31.0% overall work productivity loss (data available for 34 patients), and 33.4% activity impairment due to HAE (data available for all 47 patients with paired caregiver data). This suggests that approximately a third of the patients’ time during the past 7 days was negatively impacted due to HAE. Comparison of patient-reported HRQoL between the 47 patients with paired caregiver data and the overall 260 patient cohort indicated similar scores in the two groups (SF-12 Physical Component Summary score [mean ± SD] 45.2 ± 9.4 and 45.9 ± 9.2, respectively; SF-12 Mental Component Summary score [mean ± SD] 43.2 ± 11.5 and 42.9 ± 11.8, respectively).

#### QoL burden on caregivers of adult patients

**Fig. 4:**
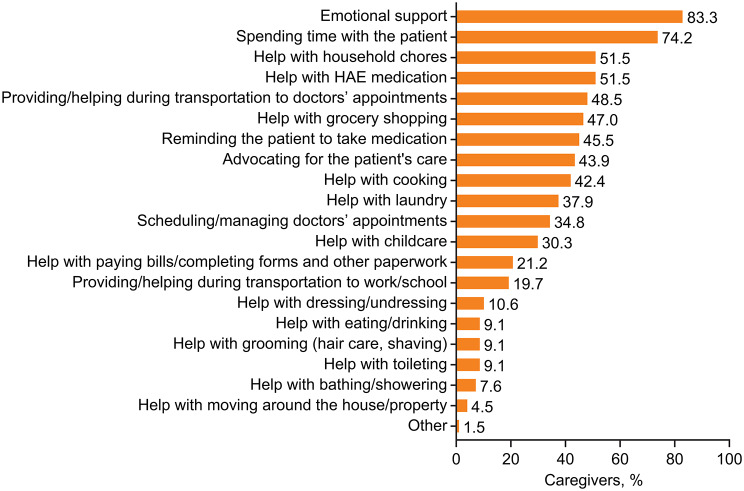
Type of care typically provided by caregivers of adult patients with HAE. *HAE* hereditary angioedema

**Fig. 5:**
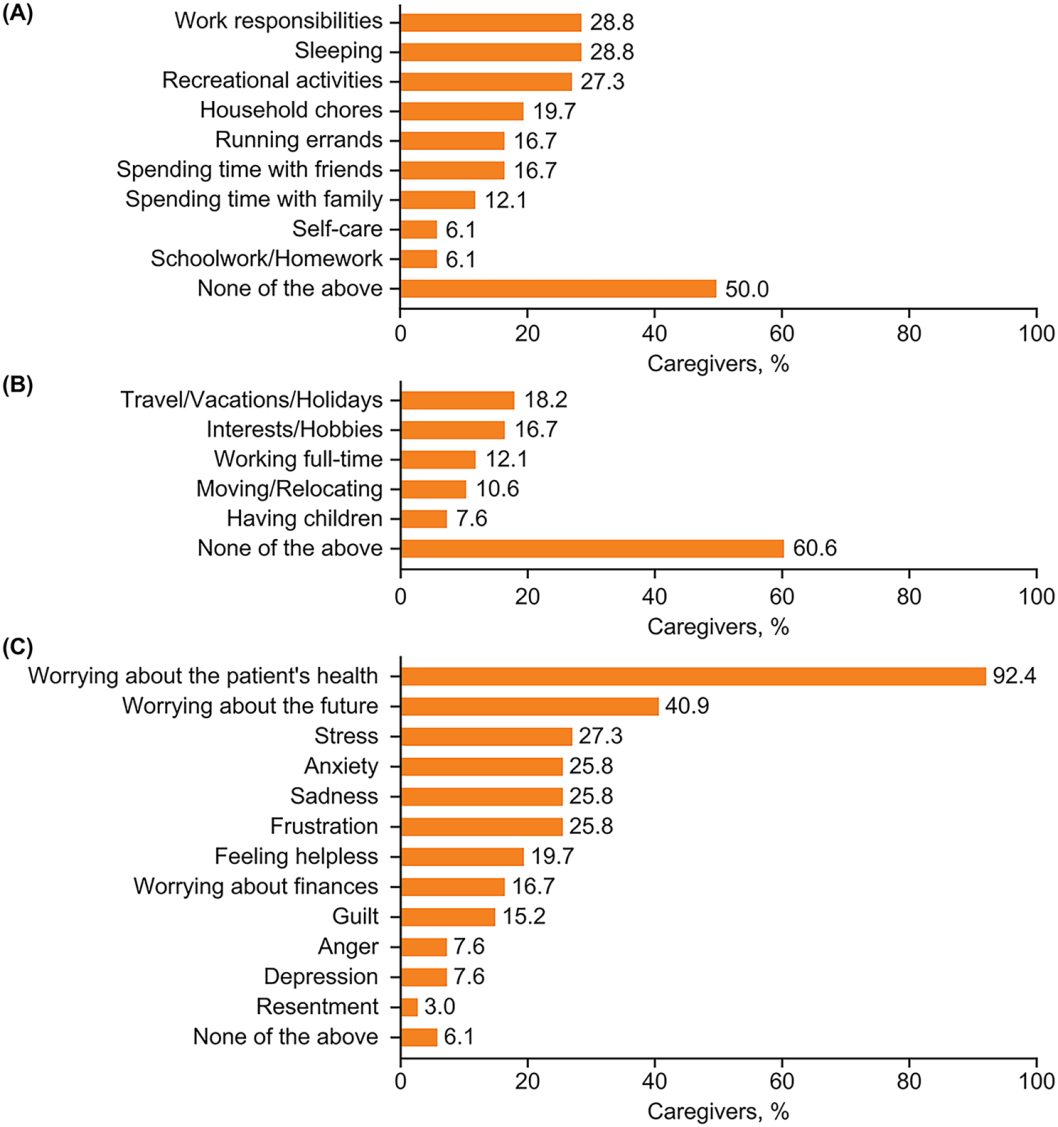
Self-reported impact on (**A**) activities (**B**) future planning and (**C**) emotional well-being of caregiving to adult patients with HAE. *HAE* hereditary angioedema

Caregivers of adult patients identified their primary role as providing emotional support and spending time with the patient; types of help usually provided to the patient are summarized in detail in Fig. 4 (data by country are summarized in Additional file 2: Supplementary Table 9). During the patient’s most recent HAE attack, help provided by the caregivers primarily included providing emotional support (72.7%), spending time with the patient (68.2%), help with administering HAE medication (51.5%), and help with household chores (48.5%). Caregiving was felt to adversely impact multiple aspects of the caregivers’ personal lives, including their work responsibilities, sleep, and recreational activities (approximately 30% for each) (Fig. 5A; data by country are provided in Additional file 2: Supplementary Table 10). The impact of caregiving in the workplace was reflected in missed days of work (24.2%), reduced working hours (7.6%), lowered career expectations (3.0%), as well as resignation from employment, acceptance of a position of lower seniority/responsibility, being denied promotion, and/or reduced salary expectations (1.5% each). In the 4 weeks before the survey, caregivers (*n* = 33) reported missing 0.94 ± 1.39 (mean ± SD) days of work (range 0–5.4 days) because of their caregiver responsibilities.

Caregivers of adult patients additionally indicated that their caregiving responsibilities adversely impacted their ability to plan their own lives, most notably their freedom to travel or go on holiday, to pursue their personal interests and hobbies, to work full time, to relocate to another city or country, and to have children (Fig. 5B; data by country are provided in Additional file 2: Supplementary Table 11). Caregivers reported a substantial impact on their emotional well-being due to worry about the patient’s health and future (Fig. 5C; data by country are provided in Additional file 2: Supplementary Table 12).

**Fig. 6:**
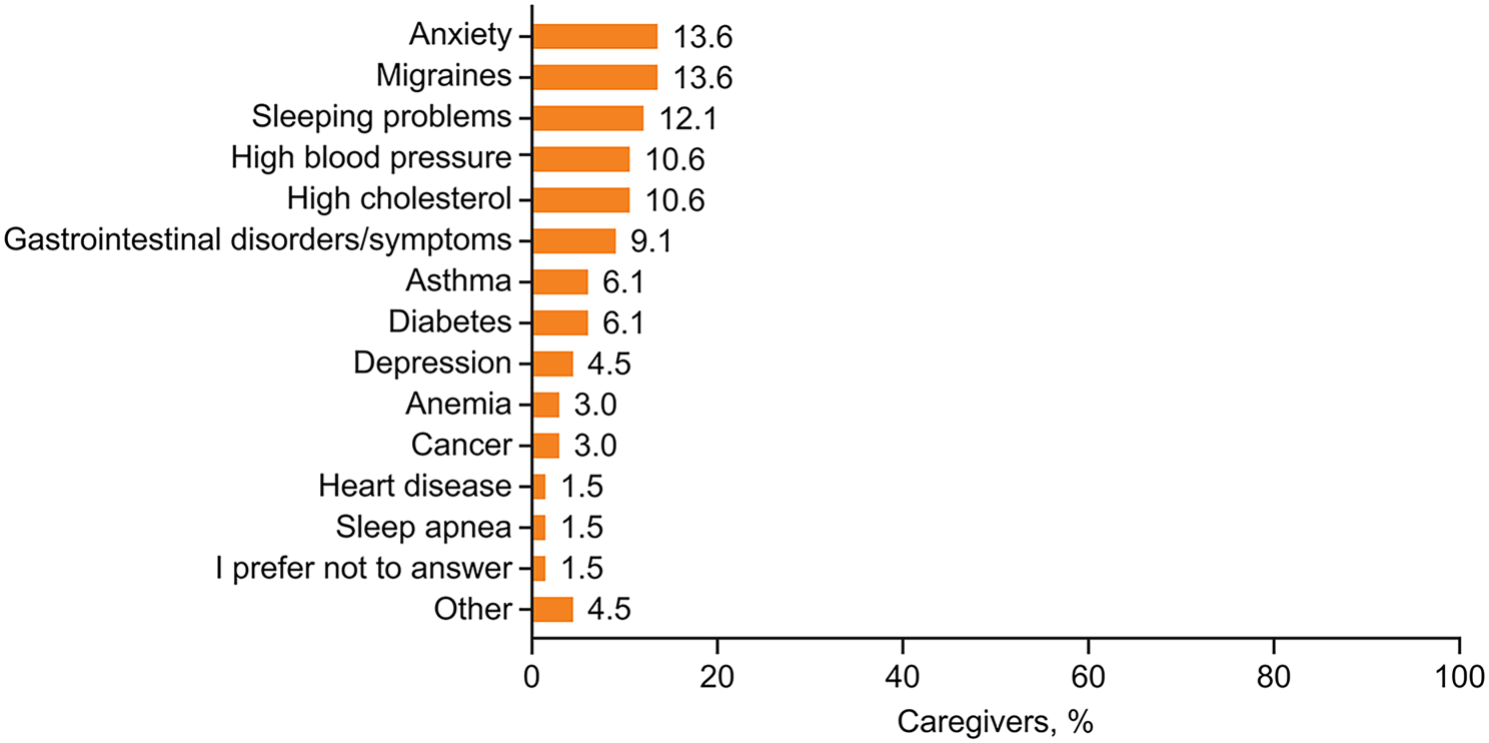
Self-reported health conditions in caregivers of adult patients with HAE

Approximately half (51.5%) of caregivers looking after adult patients reported that they themselves experienced health conditions, including anxiety, migraines, and sleep problems (over 10% of participants for each) (Fig. 6). In the last 4 weeks, 30.3% of caregivers of adults reported that they felt sadness due to patient’s HAE some, most, or all of the time; 33.3% reported spending some, most, or all of the time feeling stress or worry about the patient’s HAE, and 15.2% reported having “not at all” or “a little” time to themselves while caring for an adult with HAE.

## Discussion

This multinational real-world study was undertaken to gain the caregiver’s perspective on the emotional and physical demands of providing support to pediatric and adult patients with HAE. Two international web-based surveys, involving a total of 120 caregivers across Europe and South America, indicated that the caregivers typically lived in the same household as the patient, were relatives of the patient, and were employed; nearly half of caregivers caring for a pediatric patient had HAE themselves, whereas those caring for adult patients were generally healthy.

In the 6 months before the survey, caregivers reported a high frequency of HAE attacks among adult patients (a mean of 13.1 attacks per patient), and in children, the attack frequency was notably higher among those aged ≥ 12 years compared to those aged < 12 years (a mean of 9.1 vs. 2.7 attacks per patient). Moreover, an appreciable proportion of the HAE attacks ever experienced in children (approximately 15%) were potentially life-threatening, affecting the throat/larynx. This high rate of pediatric morbidity was reflected in substantial absenteeism from school (on average, 4 days were missed per 4 weeks) and necessitated virtually daily caregiver activity (on average, almost 24 days per 4 weeks). Of particular concern in light of the guideline recommendation that all HAE patients have on-demand treatment readily available within the home, the survey revealed that the caregivers of pediatric patients lacked a prescription for on-demand medication or long-term prophylaxis to manage HAE attacks for a substantial proportion of their children (21.8% and 58.2%, respectively). Furthermore, caregivers reported the use of first-line LTP medications (lanadelumab or C1INH) for only 2 pediatric patients. Reasons why patients were not prescribed on-demand or LTP medications were not collected in the survey, which might be considered in future research investigating barriers for access to effective treatments.

This study includes caregivers from a diverse range of countries, where regional disparities with regard to treatment access, social support and workplace policies are likely to impact the caregiver experience. With regard to treatment access, although modern LTPs such as lanadelumab and berotralstat have received approval in many countries, the relatively low rates of use might be related to issues surrounding reimbursement. Discussions around reimbursement are often multi-staged and can take some time post approval [32], and a survey of HAE-expert physicians in September 2023 found that 73% and 51% cited treatment costs and reimbursement as treatment-related unmet needs for patients [33]. Additionally, reimbursement is often limited to patients with certain disease characteristics or treatment histories. For example, in Poland reimbursement for lanadelumab was only approved in 2021 for a group of approximately 50 patients and valid until 2023 [34]. Similarly, in Ireland reimbursement of berotralstat or lanadelumab was only approved in patients with >2 attacks per week over 8 weeks despite oral LTP treatments and that those attacks were deemed not suitable for on-demand treatment with C1INH or icatibant [35].

Caring for pediatric and adult patients with HAE—a role that falls predominantly upon females in the pediatric setting but is more evenly shared between the sexes in the adult setting—was characterized as being demanding on the caregiver’s time. Care activities typically included providing emotional and supervisory support and attention within the home; scheduling, facilitating and (in the case of children) attending clinic visits; administering medication; and helping patients with domestic tasks. The most frequently reported burden attached to caring for patients was emotional concern about their health (identified as an issue by approximately 90% of caregivers) and (particularly for children) the future. Emotional burden was in many cases exacerbated by a lack of understanding of the heavy time demands placed on caregivers of pediatric patients with HAE from the school, workplace, family, and friends. Moreover, the burden of caregiving frequently extended to include detrimental effects on family relationships, social activities, and the caregiver’s own physical and mental health, as well as restrictions on the caregiver’s ability to plan their own life, from travel to education, to full-time employment, and even to having children. While caring for adult patients was reported to have lesser impact on relationships and social activities, health concerns were similarly frequent among caregivers of adult and pediatric patients. Concern for their patients’ health, combined with worry and stress about the future, affected high proportions of caregivers of pediatric and adult patients alike.

The study findings extend the limited available published information on the impact of HAE on the caregiver by using a multinational sample and distinguishing between adult and pediatric caregivers. Research to date has been confined mainly to small-scale surveys involving caregivers of adult patients and has variously considered the effect of HAE on the caregiver [20, 24, 36] and the patient’s perspective of caregiver burden [17]. In the United States, an online 2020 survey of 30 adults (predominantly immediate family members) providing informal care for an individual with HAE identified various psychosocial burdens related to HAE caregiving, including missing days of work or school and concern about the patient’s well-being [24]. Nevertheless, caregivers’ perception of their personal level of psychological well-being (mean total WHO-5 score of 64.1) was only marginally lower than that of the general population (mean total WHO-5 score of 68.7) and notably higher than the specified threshold for poor personal well-being (total WHO-5 score of 50) [24, 37, 38]. A cross-sectional survey undertaken to assess healthcare resource utilization among patients with HAE in Spain, Germany, and Denmark additionally provided information on the impact of HAE on caregiver productivity [36]. For those caregivers in employment, absenteeism increased in line with the frequency and severity of HAE attacks: over a 6-month period, caregivers lost, on average, 3.3, 3.6, and 10.1 days of work on account of caring for patients with HAE attacks occurring less than once a month, at least once a month but less than once a week, and at least once a week, respectively [36].

The SPRING Study, which assessed the safety and efficacy of lanadelumab in patients aged 2 to < 12 years with HAE, also provided an indication of the effect of HAE on the child’s caregiver/parents/family, as evaluated with the Family Impact Module (FIM) of the Parent or Caregiver Proxy Report of Pediatric Quality of Life Inventory (PedsQL) [39]. Marked improvements in parent HRQoL summary score, family functioning summary score, and PedsQL-FIM total score were reported during the study [39].

Interestingly, a similar burden has been reported in carers of patients with other chronic, episodic conditions. For example, a qualitative study of family carers of patients with atopic dermatitis or psoriasis found that the main contributing factors to carer burden included worries about the patients’ future, impact on sleep, leisure activities and daily activities, and a lack of understanding about the patients’ condition [40]. Similarly, a review of qualitative studies on the burden of caregiving for patients with epilepsy found that anxiety, concerns about the patients’ future, missing work and missing out on socializing all impacted the burden felt by caregivers [41]. Additionally, in a study of 200 caregivers to patients with multiple sclerosis, 68% reported a significant burden with uncertainty about the patients’ future the largest contributor [42]. Taken together, these findings suggest there may be similar influences on the burden experienced by carers of patients with chronic conditions.

Strengths of the present study include its large sample size relative to previous investigations into the impact of HAE on the caregivers, and its recruitment of caregivers from countries with different treatment approaches and differing access to long-term prophylactic medication. Limitations of the study include uncertainty regarding the generalizability of its findings, the potential for selection bias when recruiting caregivers through patient advocacy organizations, and the potential for recall bias in self-reported, retrospective data. ‘Caregiver’ is a somewhat heterogenous term, and it is likely that those self-reporting as such will have a range of different roles and responsibilities. In addition, a cross-sectional survey is unable to establish a causal relationship between caregiver role and caregiver burden. Additionally, caregiver recruitment in certain countries was low; in both of the surveys, the majority of caregivers were from countries in South America (Argentina, Brazil, and Colombia; 35 of 54 caregivers of children and 46 of 66 caregivers of adults), with caregivers from countries in Europe being underrepresented in these surveys. Differences in cultural values, family support systems, public health systems, and access to treatment are likely to affect the way in which caregivers in different countries perceive their roles and burdens. A previous study also suggested that, compared with caregivers without HAE, caregivers who have HAE themselves are likely to perceive a higher level of well-being for individuals with HAE for whom they provide care [24]. Moreover, the study provided no quantitative assessment of how the burden of caregiving for HAE affects the caregivers’ quality of life. Carers’ burden of disease was assessed using a bespoke survey tailored to HAE; the use of validated tools to assess caregiver burden in future research would enable comparisons between carers of patients with HAE and carers in a wider general population.

## Conclusions

The role of being a caregiver to a patient with HAE is highly time-demanding and adversely impacts various facets of a caregiver’s life and in particular their emotional wellbeing, with a large proportion of caregivers reporting worry about the future. Other potential challenges faced by the caregiver include restrictions on social activities, personal relationships, and employment, as well as an impact on their own health.

## Supplementary Information

Below is the link to the electronic supplementary material.

Supplementary Material 1: Additional file 1: 2-page visual summary

Supplementary Material 2: Additional file 2: Supplementary Tables 1–12. Supplementary Table 1 Caregiver-reported demographic and clinical characteristics of pediatric patients with HAE by country. Supplementary Table 2 Caregiver-reported demographic and clinical characteristics of pediatric patients with HAE by age group. Supplementary Table 3 Areas of the body affected by any HAE attack ever experienced in pediatric patients with HAE. Supplementary Table 4 Type of care provided by caregivers of pediatric patients with HAE by country. Supplementary Table 5 Self-reported impact on daily activities of caregiving to pediatric patients with HAE by country. Supplementary Table 6 Self-reported impact on relationships of caregiving to pediatric patients with HAE by country. Supplementary Table 7 Self-reported impact on emotional well-being of caregiving to pediatric patients with HAE by country. Supplementary Table 8 Caregiver-reported clinical characteristics of adult patients with HAE by country. Supplementary Table 9 Type of care typically provided by caregivers of adult patients with HAE. Supplementary Table 10 Self-reported impact on daily activities of caregiving to adult patients with HAE by country. Supplementary Table 11 Self-reported impact on future planning of caregiving to adult patients with HAE by country. Supplementary Table 12 Self-reported impact on emotional well-being of caregiving to adult patients with HAE by country

## Data Availability

The datasets generated during and/or analyzed during the current study are available from the corresponding author on reasonable request.
