# Supplementary material for: Burden of hereditary angioedema: results from a multinational survey of caregivers for adult and pediatric patients
Source: Orphanet J Rare Dis. 2026 Feb 12;21:55. doi: 10.1186/s13023-025-04123-2 (PMC12895942; doi:10.1186/s13023-025-04123-2)
Supplement: Supplementary file 1 — Supplementary Material 1: Additional file 1: 2-page visual summary [file 13023_2025_4123_MOESM1_ESM.pdf]

Burden of hereditary angioedema: results from a multinational survey of patients' caregivers

This is a visual summary of an article titled "Burden of hereditary angioedema: results from a multinational survey of caregivers for adult and pediatric patients," which was published in *Orphanet Journal of Rare Diseases* in 2025.

How to say:

- Hereditary: "hr-eh-duh-the-ree"
- Angioedema: "an-jee-ow-uh-dee-muh"

What is hereditary angioedema (HAE for short)?

HAE is a rare **genetic disease** that impacts around 1 in 50,000 to 1 in 10,000 individuals. HAE causes painful swellings of the skin and deeper tissues, also known as HAE attacks.

How often HAE attacks occur, how severe they are, and what causes them can be unpredictable. Swelling is the most common in the face, throat, abdomen, hands, genitals, and feet. Any attack can be painful, and an attack affecting the airway can become life-threatening.

- Most people with HAE have their first attack as a child or a teenager, and attacks recur throughout life.

What is the role of the caregiver for people with HAE?

HAE attacks can disrupt the day-to-day activities, school/work performance, and well-being of the person with HAE.

Many people with HAE have an informal caregiver or carer—often a family member—who helps them cope with everyday tasks.

- Previous studies have looked into the impact of HAE in individuals living with the disease in some countries, but there is less information available about the impact of HAE on caregivers.

Why was the study done?

The funder of the study, Takeda, wanted to better understand how looking after a child or adult with HAE affects caregivers in different countries.

What were the questions that researchers wanted to answer?

- What type of help do people living with HAE get from caregivers?
- How does providing care for someone with HAE affect the caregiver's health and personal life?

What was the study plan?

The study took place between **July 2022** and **February 2023** in **13 countries** in **Europe** and **South America**. **Two separate online surveys** were performed: one for **caregivers of children with HAE** (younger than 18 years) and one for caregivers of **adults with HAE.**

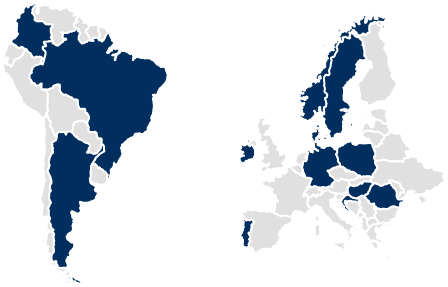

The surveys were conducted in

- Argentina
- Brazil
- Colombia
- Croatia
- Denmark
- Germany
- Hungary
- Ireland
- Norway
- Poland
- Portugal
- Romania
- Sweden

54 caregivers of children and **66** caregivers of adults completed the web-based survey

All caregivers were providing care to people with HAE on an unpaid voluntary basis.

The caregivers answered survey questions about their own health and that of the people with HAE they provide care for. The caregivers also described the effects of HAE on themselves and their people with HAE.

What were the main results of the two surveys?

Caregivers of children with HAE had an average age of 41 years.

Caregivers of adults with HAE had an average age of 43 years and were evenly split between men and women.

Most caregivers of children with HAE were women.

80%

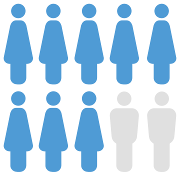

Almost half of caregivers of children with HAE had HAE themselves.

46%

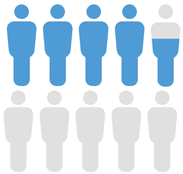

Most caregivers of adults with HAE lived in the same household as the adult with HAE.

76%

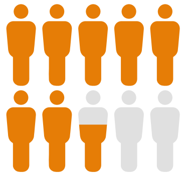

Over one-half of the caregivers of adults with HAE were the spouse/partner of the adult with HAE.

55%

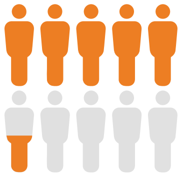

Most caregivers of people with HAE were employed outside the home

Caregivers of children

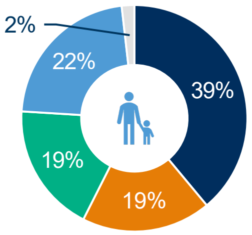

Caregivers of adults

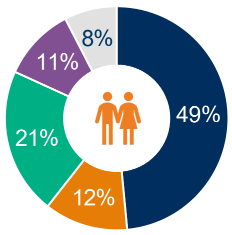

- Full-time work
- Part-time work
- Self-employed
- Stay-at-home
- Retired/stay-at-home
- Other

What did caregivers consider was their main role?

Caregivers of children with HAE

- Spending time with their child **85%**
- Managing doctor's appointments **83%**
- Emotional support **82%**
- Transportation to/from doctors' appointments **76%**
- Transportation to/from school/work/other activities **72%**
- Advocating for the child's care **65%**

On average, caregivers of children with HAE provided care for 23.5 days each month

Caregivers of adults with HAE

- Emotional support **83%**
- Spending time with the adult with HAE **74%**
- Help with household chores **52%**
- Help with HAE medications **52%**
- Providing/helping during transportation to doctors' appointments **49%**
- Help with grocery shopping **47%**

On average, caregivers of adults with HAE provided care for 14.4 days each month

The caregiver main role and time requirement for caregiving differed due to different needs for child and adult care.

How did caring for children with HAE affect the caregivers' quality of life?

Caregivers of children with HAE felt that family, friends, and work colleagues did not appreciate the amount of time they spent providing care. Providing care especially impacted their work, sleep, and household chores, and the amount of time they were able to spend with family and friends .

- Work **37%**
- Sleep **37%**
- Housework **32%**
- Time with friends **24%**
- Time with family **30%**

How did caring for adults with HAE affect the caregivers' quality of life?

Caregivers of adults with HAE felt that caregiving interfered with their work and sleep, and their freedom to plan holidays, work full-time, relocate, and have children.

- Work **29%**
- Sleep **29%**
- Holidays **18%**
- Work full-time **12%**
- Relocate **11%**
- Have children **8%**

What does this study tell us and why is it important?

This study shows that the caregiver role in HAE is time-demanding. Caring for someone with HAE can have a negative effect on the caregiver's employment opportunities, social activities, personal relationships, and ability **to plan their own life**. Caregiver burden also varies based on whether they provide care for children or adults with HAE. Time commitment for looking after children with HAE is higher than that for looking after adults with HAE.

More information

More information about HAE can be found on the websites below:

HAE International: https://haei.org/

Genetic and Rare Diseases Information Center: https://rarediseases.info.nih.gov/diseases/5979/hereditary-angioedema

The study was funded by Takeda Development Center Americas, Inc. Medical writing support for this summary was provided by Milda Tyler, PhD, of Excel Scientific Solutions, Inc.
The original authors of the full article reviewed and approved this summary.
