# Supplementary material for: Burden of hereditary angioedema: results from a multinational survey of caregivers for adult and pediatric patients
Source: Orphanet J Rare Dis. 2026 Feb 12;21:55. doi: 10.1186/s13023-025-04123-2 (PMC12895942; doi:10.1186/s13023-025-04123-2)
Supplement: Supplementary file 2 — Supplementary Material 2: Additional file 2: Supplementary Tables 1–12. Supplementary Table 1 Caregiver-reported demographic and clinical characteristics of pediatric patients with HAE by country. Supplementary Table 2 Caregiver-reported demographic and clinical characteristics of pediatric patients with HAE by age group. Supplementary Table 3 Areas of the body affected by any HAE attack ever experienced in pediatric patients with HAE. Supplementary Table 4 Type of care provided by caregivers of pediatric patients with HAE by country. Supplementary Table 5 Self-reported impact on daily activities of caregiving to pediatric patients with HAE by country. Supplementary Table 6 Self-reported impact on relationships of caregiving to pediatric patients with HAE by country. Supplementary Table 7 Self-reported impact on emotional well-being of caregiving to pediatric patients with HAE by country. Supplementary Table 8 Caregiver-reported clinical characteristics of adult patients with HAE by country. Supplementary Table 9 Type of care typically provided by caregivers of adult patients with HAE. Supplementary Table 10 Self-reported impact on daily activities of caregiving to adult patients with HAE by country. Supplementary Table 11 Self-reported impact on future planning of caregiving to adult patients with HAE by country. Supplementary Table 12 Self-reported impact on emotional well-being of caregiving to adult patients with HAE by country [file 13023_2025_4123_MOESM2_ESM.docx]

Additional file 2: Supplementary Tables 1–12

**Burden of hereditary angioedema: results from a multinational survey of caregivers for adult and pediatric patients**

Inmaculada Martinez-Saguer,^1^ Anete S. Grumach,^2^ Ricardo Zwiener,^3^ Mauricio Sarrazola,^4^ Angela Simon,^5^ Ryan Murphy,^6^ Marie De La Cruz,^6^ Maureen Watt^5^

^1^HZRM Hemophilia Center Rhine Main, Frankfurt/Main; Germany; ^2^Clinical Immunology, Faculdade de Medicina, Centro Universitario Faculdade de Medicina ABC (CEUFMABC), Santo Andre, Brazil; ^3^Servicio de Alergia e Inmunología Clínica, Hospital Universitario Austral, Pilar, Buenos Aires, Argentina; ^4^Departamento de Medicina, Grupo GIPPAM, Universidad de Pamplona, Cúcuta, Colombia; ^5^Takeda Development Center Americas Inc, Lexington, MA, USA; ^6^ICON, Raleigh, NC, USA

**Correspondence:**

**Name:** Maureen Watt

**Address:** Takeda Development Center Americas, Inc., 500 Kendall Street, Cambridge, MA 02142, USA

**Tel:** +1 617 784 9707

**E-mail:** [maureen.watt@takeda.com](mailto:maureen.watt@takeda.com)

**Supplementary Table 1** Caregiver-reported demographic and clinical characteristics of pediatric patients with HAE by country

| Characteristic | **ARG**  n=19 | **BRA**  n=7 | **COL**  n=10 | **CRO**  n=1 | **DEN**  n=2 | **GER**  n=2 | **HUN**  n=5 | **IRL**  n=1 | **POL**  n=6 | **POR**  **n=2** | **Total**  N=55 |
| --- | --- | --- | --- | --- | --- | --- | --- | --- | --- | --- | --- |
| Sex, n (%) |  |  |  |  |  |  |  |  |  |  |  |
| Female | 9 (47.4) | 3 (42.9) | 6 (60.0) | 1 (100) | 0 (0.0) | 1 (50.0) | 2 (40.0) | 1 (100) | 4 (66.7) | 1 (50.0) | 28 (50.9) |
| Male | 10 (52.6) | 4 (57.1) | 4 (40.0) | 0 (0.0) | 2 (100) | 1 (50.0) | 3 (60.0) | 0 (0.0) | 2 (33.3) | 1 (50.0) | 27 (49.1) |
|  |  |  |  |  |  |  |  |  |  |  |  |
| Age, years |  |  |  |  |  |  |  |  |  |  |  |
| Mean ± SD | 11.3 ± 3.9 | 11.6 ± 4.4 | 10.7 ± 2.6 | NR^a^ | 14.5 ± 0.7 | 15.5 ± 0.7 | 6.0 ± 3.6 | NR^a^ | 9.0 ± 3.2 | 10.0 ± 1.4 | 10.6 ± 3.8 |
| Median | 12.0 | 14.0 | 11.0 | NR^a^ | 14.5 | 15.5 | 5.0 | NR^a^ | 8.5 | 10.0 | 11.0 |
| IQR | 7.0–15.0 | 7.0–14.0 | 8.0–13.0 | NR^a^ | NR^a^ | NR^a^ | 5.0–9.0 | NR^a^ | 8.0–12.0 | NR^a^ | 8.0–14.0 |
| Range | 5.0–16.0 | 5.0–17.0 | 7.0–15.0 | NR^a^ | NR^a^ | NR^a^ | 1.0–10.0 | NR^a^ | 4.0–13.0 | NR^a^ | 1.0–17.0 |
| Type of HAE, n (%) |  |  |  |  |  |  |  |  |  |  |  |
| HAE-C1INH-Type1 | 16 (84.2) | 3 (42.9) | 4 (40.0) | 1 (100.0) | 2 (100.0) | 2 (100.0) | 2 (40.0) | 0 | 5 (83.3) | 2 (100.0) | 37 (67.3) |
| HAE-C1INH-Type2 | 1 (5.3) | 1 (14.3) | 0 | 0 | 0 | 0 | 1 (20.0) | 1 (100.0) | 0 | 0 | 4 (7.3) |
| HAE-nC1INH | 2 (10.5) | 0 | 0 | 0 | 0 | 0 | 0 | 0 | 0 | 0 | 2 (3.6) |
| HAE-C1INH undifferentiated^b^ | 0 | 3 (42.9) | 5 (50.0) | 0 | 0 | 0 | 2 (40.0) | 0 | 1 (16.7) | 0 | 11 (20.0) |
| Unknown^c^ | 0 | 0 | 1 (10.0) | 0 | 0 | 0 | 0 | 0 | 0 | 0 | 1 (1.8) |
| Age at HAE onset, years |  |  |  |  |  |  |  |  |  |  |  |
| Mean ± SD | 6.3 ± 4.8 | 5.3 ± 3.5 | 3.2 ± 2.2 | NR^a^ | 4.0 ± 1.4 | 7.0 ± 7.1 | 1.0 ± 1.4 | NR^a^ | 4.2 ± 2.6 | NR^a^ | 4.5 ± 3.9 |
| Median | 5.0 | 6.0 | 3.0 | NR^a^ | NR^a^ | NR^a^ | 0.0 | NR^a^ | 3.5 | NR^a^ | 4.0 |
| Range | 0.0–16.0 | 1.0–11.0 | 0.0–7.0 | NR^a^ | NR^a^ | NR^a^ | 0.0–3.0 | NR^a^ | 2.0–9.0 | NR^a^ | 0.0–16.0 |
| Age at HAE diagnosis, years |  |  |  |  |  |  |  |  |  |  |  |
| Mean ± SD | 6.5 ± 4.3 | 3.7 ± 2.4 | 4.6 ± 2.8 | NR^a^ | NR^a^ | NR^a^ | 0.2 ± 0.5 | 3.0 | 2.7 (1.9) | 2.5 (3.5) | 4.2 (3.6) |
| Median | 5.0 | 4.0 | 4.5 | NR^a^ | NR^a^ | NR^a^ | 0.0 | 3.0 | 3.0 | NR^a^ | 4.0 |
| Range | 0.0–14.0 | 1.0–6.0 | 0.0–9.0 | NR^a^ | NR^a^ | NR^a^ | 0.0–1.0 | 3.0–3.0 | 0.0–5.0 | NR^a^ | 0.0–14.0 |
| Doctors seen before HAE diagnosis, n |  |  |  |  |  |  |  |  |  |  |  |
| Mean ± SD | 1.6 ± 1.3 | 1.3 ± 0.8 | 3.6 ± 2.8 | 3.0 | 1.0 ± 0.0 | 1.0 ± 0.0 | 1.0 ± 0.0 | 1.0 | 1.5 ± 1.2 | 2.0 ± 0.0 | 1.8 ± 1.7 |
| Median | 1.0 | 1.0 | 3.5 | 3.0 | 1.0 | 1.0 | 1.0 | 1.0 | 1.0 | 2.0 | 1.0 |
| Range | 1.0–5.0 | 1.0–3.0 | 1.0–10.0 | 3.0–3.0 | 1.0–1.0 | 1.0–1.0 | 1.0–1.0 | 1.0–1.0 | 1.0–4.0 | 2.0–2.0 | 1.0‒10.0 |

All data presented in this table are self-reported by survey participants

*ARG* Argentina, *BRA* Brazil, *COL* Colombia, *CRO* Croatia, *DEN* Denmark, *GER* Germany, *HAE* hereditary angioedema, *HAE-C1INH* hereditary angioedema due to C1 inhibitor deficiency; *HAE-nC1INH* hereditary angioedema due to normal C1 inhibitor, *HUN* Hungary, *IRL* Ireland, *IQR,* interquartile range, *POL* Poland, *POR* Portugal, *SD* standard deviation

^a^Data not reported to prevent person reidentification

^b^Includes patients for whom the caregiver selected the answer “Unsure of exact HAE type, but it is either HAE Type I or II” to the survey question “Which type of HAE does the patient have?”

^c^Includes patients for whom the caregiver selected the answer “I don’t know what type of HAE” to the survey question “Which type of HAE does the patient have?”

**Supplementary Table 2** Caregiver-reported demographic and clinical characteristics of pediatric patients with HAE by age group

| Characteristic | **<12 years**  n=30 | **≥12 years**  n=25 | **Total**  N=55 |
| --- | --- | --- | --- |
| Sex, n (%) |  |  |  |
| Female | 14 (46.7) | 14 (56.0) | 28 (50.9) |
| Male | 16 (53.3) | 11 (44.0) | 27 (49.1) |
| Age, years |  |  |  |
| Mean ± SD | 7.6 ± 2.4 | 14.1 ± 1.5 | 10.6 ± 3.8 |
| Median | 8.0 | 14.0 | 11.0 |
| IQR | 6.0–9.0 | 13.0–15.0 | 8.0–14.0 |
| Range | 1.0–11.0 | 12.0–17.0 | 1.0–17.0 |
| Type of HAE, n (%) |  |  |  |
| HAE-C1INH-Type1 | 16 (53.3) | 21 (84.0) | 37 (67.3) |
| HAE-C1INH-Type2 | 4 (13.3) | 0 | 4 (7.3) |
| HAE-nC1INH | 0 | 2 (8.0) | 2 (3.6) |
| HAE-C1INH undifferentiated^a^ | 10 (33.3) | 1 (4.0) | 11 (20.0) |
| Unknown^b^ | 0 | 1 (4.0) | 1 (1.8) |
| Age at HAE onset, years |  |  |  |
| Mean ± SD | 2.7 ± 2.2 | 6.6 ± 4.5 | 4.5 ± 3.9 |
| Median | 2.0 | 6.0 | 4.0 |
| Range | 0.0–9.0 | 0.0–16.0 | 0.0–16.0 |
| Age at HAE diagnosis, years |  |  |  |
| Mean ± SD | 3.3 ± 2.4 | 5.2 ± 4.6 | 4.2 ± 3.6 |
| Median | 4.0 | 3.0 | 4.0 |
| Range | 0.0–8.0 | 0.0–14.0 | 0.0–14.0 |
| Doctors seen before HAE diagnosis, n |  |  |  |
| Mean ± SD | 1.9 ± 1.9 | 1.8 ± 1.4 | 1.8 ± 1.7 |
| Median | 1.0 | 1.0 | 1.0 |
| Range | 1–10 | 1–5 | 1–10 |

All data presented in this table are self-reported by survey participants

*HAE* hereditary angioedema, *HAE-C1INH* hereditary angioedema due to C1 inhibitor deficiency; *HAE-nC1INH* hereditary angioedema due to normal C1 inhibitor, *IQR,* interquartile range, *SD* standard deviation

^a^Includes patients for whom the caregiver selected the answer “Unsure of exact HAE type, but it is either HAE Type I or II” to the survey question “Which type of HAE does the patient have?”

^b^Includes patients for whom the caregiver selected the answer “I don’t know what type of HAE” to the survey question “Which type of HAE does the patient have?”

**Supplementary Table 3** Areas of the body affected by any HAE attack ever experienced in pediatric patients with HAE

| **Area of the body, n (%)^a^** | **ARG**  **n=19** | **BRA**  **n=7** | **COL**  **n=10** | **CRO**  **n=1** | **DEN**  **n=2** | **GER**  **n=2** | **HUN**  **n=5** | **IRL**  **n=1** | **POL**  **n=6** | **POR**  **n=2** | **Total**  **N=55** |
| --- | --- | --- | --- | --- | --- | --- | --- | --- | --- | --- | --- |
| Arms (including the joints) | 2 (10.5) | 3 (42.9) | 3 (30.0) | 0 | 0 | 0 | 1 (20.0) | 0 | 0 | 2 (100.0) | 11 (20.0) |
| Legs (including the joints) | 3 (15.8) | 1 (14.3) | 2 (20.0) | 0 | 0 | 0 | 1 (20.0) | 0 | 1 (16.7) | 2 (100.0) | 10 (18.2) |
| Hands | 9 (47.4) | 6 (85.7) | 7 (70.0) | 0 | 2 (100.0) | 0 | 3 (60.0) | 1 (100.0) | 1 (16.7) | 2 (100.0) | 31 (56.4) |
| Feet | 4 (21.1) | 5 (71.4) | 5 (50.0) | 0 | 2 (100.0) | 0 | 1 (20.0) | 1 (100.0) | 1 (16.7) | 2 (100.0) | 21 (38.2) |
| Cheeks | 2 (10.5) | 0 | 3 (30.0) | 0 | 1 (50.0) | 0 | 1 (20.0) | 1 (100.0) | 3 (50.0) | 1 (50.0) | 12 (21.8) |
| Lips | 2 (10.5) | 2 (28.6) | 5 (50.0) | 0 | 0 | 1 (50.0) | 0 | 1 (100.0) | 1 (16.7) | 0 | 12 (21.8) |
| Eyes | 6 (31.6) | 2 (28.6) | 3 (30.0) | 0 | 1 (50.0) | 0 | 1 (20.0) | 0 | 1 (16.7) | 0 | 14 (25.5) |
| Ears | 1 (5.3) | 1 (14.3) | 2 (20.0) | 0 | 0 | 0 | 0 | 0 | 0 | 0 | 4 (7.3) |
| Tongue | 1 (5.3) | 0 | 1 (10.0) | 0 | 0 | 0 | 0 | 0 | 0 | 0 | 2 (3.6) |
| Uvula | 0 | 0 | 1 (10.0) | 0 | 0 | 0 | 0 | 0 | 0 | 0 | 1 (1.8) |
| Throat or larynx | 1 (5.3) | 2 (28.6) | 1 (10.0) | 0 | 1 (50.0) | 1 (50.0) | 0 | 0 | 1 (16.7) | 1 (50.0) | 8 (14.5) |
| Back | 1 (5.3) | 1 (14.3) | 1 (10.0) | 0 | 0 | 0 | 0 | 0 | 0 | 0 | 3 (5.5) |
| Side of the body (between chest and hip) | 1 (5.3) | 1 (14.3) | 1 (10.0) | 0 | 0 | 0 | 0 | 0 | 0 | 1 (50.0) | 4 (7.3) |
| Chest | 0 | 0 | 0 | 1 (100.0) | 0 | 0 | 0 | 0 | 0 | 0 | 1 (1.8) |
| Abdomen | 9 (47.4) | 3 (42.9) | 7 (70.0) | 0 | 2 (100.0) | 2 (100.0) | 1 (20.0) | 1 (100.0) | 5 (83.3) | 2 (100.0) | 32 (58.2) |
| Buttocks | 0 | 1 (14.3) | 1 (10.0) | 1 (100.0) | 0 | 0 | 1 (20.0) | 0 | 0 | 0 | 4 (7.3) |
| Genitals | 1 (5.3) | 0 | 3 (30.0) | 0 | 0 | 1 (50.0) | 1 (20.0) | 0 | 0 | 1 (50.0) | 7 (12.7) |
| Bowels or rectum | 2 (10.5) | 4 (57.1) | 0 | 0 | 1 (50.0) | 1 (50.0) | 0 | 0 | 0 | 1 (50.0) | 9 (16.4) |
| Bladder | 0 | 0 | 0 | 0 | 0 | 0 | 0 | 0 | 1 (16.7) | 0 | 1 (1.8) |
| Other | 5 (26.3) | 0 | 0 | 0 | 0 | 0 | 1 (20.0) | 0 | 1 (16.7) | 0 | 7 (12.7) |
| I don‘t know or remember | 1 (5.3) | 0 | 0 | 0 | 0 | 0 | 2 (40.0) | 0 | 0 | 0 | 3 (5.5) |

All data presented in this table are self-reported by survey participants

*ARG* Argentina, *BRA* Brazil, *COL* Colombia, *CRO* Croatia, *DEN* Denmark, *GER* Germany, *HUN* Hungary, *IRL* Ireland, *POL* Poland, *POR* Portugal

^a^The sum of categories may exceed 100%, as responses were not mutually exclusive

**Supplementary Table 4** Type of care typically provided by caregivers of pediatric patients with HAE by country

| **Type of care provided, n (%)** | **ARG**  **n=19** | **BRA**  **n=6^a^** | **COL**  **n=10** | **CRO**  **n=1** | **DEN**  **n=2** | **GER**  **n=2** | **HUN**  **n=5** | **IRL**  **n=1** | **POL**  **n=6** | **POR**  **n=2** | **Total**  **N=54** |
| --- | --- | --- | --- | --- | --- | --- | --- | --- | --- | --- | --- |
| Help with dressing or undressing | 6 (31.6) | 2 (33.3) | 1 (10.0) | 0 | 0 | 0 | 2 (40.0) | 0 | 0 | 0 | 11 (20.4) |
| Help with bathing or showering | 4 (21.1) | 2 (33.3) | 1 (10.0) | 0 | 0 | 0 | 3 (60.0) | 1 (100.0) | 1 (16.7) | 0 | 12 (22.2) |
| Help with eating or drinking | 3 (15.8) | 1 (16.7) | 0 | 0 | 0 | 0 | 2 (40.0) | 0 | 0 | 0 | 6 (11.1) |
| Help with grooming (hair care, shaving) | 5 (26.3) | 2 (33.3) | 1 (10.0) | 0 | 0 | 0 | 1 (20.0) | 1 (100.0) | 2 (33.3) | 0 | 12 (22.2) |
| Help with going to the toilet | 3 (15.8) | 1 (16.7) | 1 (10.0) | 0 | 0 | 0 | 1 (20.0) | 0 | 0 | 0 | 6 (11.1) |
| Help with moving around the house or property | 2 (10.5) | 1 (16.7) | 0 | 0 | 0 | 0 | 1 (20.0) | 0 | 0 | 0 | 4 (7.4) |
| Help with homework | 7 (36.8) | 3 (50.0) | 7 (70.0) | 0 | 1 (50.0) | 2 (100.0) | 2 (40.0) | 1 (100.0) | 2 (33.3) | 0 | 25 (46.3) |
| Home schooling | 7 (36.8) | 2 (33.3) | 5 (50.0) | 0 | 0 | 0 | 2 (40.0) | 0 | 3 (50.0) | 0 | 19 (35.2) |
| Help with administering HAE medication | 9 (47.4) | 3 (50.0) | 7 (70.0) | 1(100.0) | 2 (100.0) | 1 (50.0) | 1 (20.0) | 1 (100.0) | 4 (66.7) | 1 (50.0) | 30 (55.6) |
| Reminding patient to take medications | 7 (36.8) | 3 (50.0) | 5 (50.0) | 0 | 1 (50.0) | 0 | 0 | 0 | 0 | 2 (100.0) | 18 (33.3) |
| Scheduling or managing doctors appointments | 17 (89.5) | 3 (50.0) | 8 (80.0) | 0 | 2 (100.0) | 2 (100.0) | 4 (80.0) | 1 (100.0) | 6 (100.0) | 2 (100.0) | 45 (83.3) |
| Emotional support | 17 (89.5) | 3 (50.0) | 9 (90.0) | 0 | 1 (50.0) | 2 (100.0) | 5 (100.0) | 1 (100.0) | 4 (66.7) | 2 (100.0) | 44 (81.5) |
| Spending time with your child | 18 (94.7) | 4 (66.7) | 8 (80.0) | 0 | 1 (50.0) | 2 (100.0) | 5 (100.0) | 1 (100.0) | 5 (83.3) | 2 (100.0) | 46 (85.2) |
| Advocating for the child’s care | 14 (73.7) | 2 (33.3) | 7 (70.0) | 0 | 2 (100.0) | 2 (100.0) | 5 (100.0) | 1 (100.0) | 2 (33.3) | 0 | 35 (64.8) |
| Providing transportation to and from school, work, or other activities | 15 (78.9) | 4 (66.7) | 7 (70.0) | 0 | 1 (50.0) | 0 | 5 (100.0) | 1 (100.0) | 4 (66.7) | 2 (100.0) | 39 (72.2) |
| Providing transportation to and from doctors appointments | 14 (73.7) | 3 (50.0) | 7 (70.0) | 0 | 2 (100.0) | 2 (100.0) | 5 (100.0) | 1 (100.0) | 5 (83.3) | 2 (100.0) | 41 (75.9) |
| Other | 1 (5.3) | 0 | 0 | 0 | 0 | 0 | 0 | 0 | 0 | 0 | 1 (1.9) |

All data presented in this table are self-reported by survey participants

*ARG* Argentina, *BRA* Brazil, *COL* Colombia, *CRO* Croatia, *DEN* Denmark, *GER* Germany, *HUN* Hungary, *IRL* Ireland, *POL* Poland, *POR* Portugal

^a^One caregiver from Brazil filled the survey for two children; the caregiver was not asked questions about types of care provided twice

**Supplementary Table 5** Self-reported impact on daily activities of caregiving to pediatric patients with HAE by country

| **Activities Impacted, n (%)** | **ARG**  **n=19** | **BRA**  **n=6** | **COL**  **n=10** | **CRO**  **n=1** | **DEN**  **n=2** | **GER n=2** | **HUN**  **n=5** | **IRL**  **n=1** | **POL**  **n=6** | **POR**  **n=2** | **Total**  **N=54^a^** |
| --- | --- | --- | --- | --- | --- | --- | --- | --- | --- | --- | --- |
| Self-care | 5 (26.3) | 1 (16.7) | 1 (10.0) | 0 | 0 | 0 | 0 | 1 (100.0) | 0 | 0 | 8 (14.8) |
| Recreational activities | 8 (42.1) | 2 (33.3) | 2 (20.0) | 1 (100.0) | 0 | 2 (100.0) | 1 (20.0) | 0 | 0 | 0 | 16 (29.6) |
| Household chores | 6 (31.6) | 3 (50.0) | 5 (50.0) | 0 | 0 | 1 (50.0) | 0 | 1 (100.0) | 1 (16.7) | 0 | 17 (31.5) |
| Work responsibilities | 8 (42.1) | 2 (33.3) | 4 (40.0) | 1 (100.0) | 1 (50.0) | 1 (50.0) | 0 | 0 | 2 (33.3) | 1 (50.0) | 20 (37.0) |
| Running errands | 5 (26.3) | 1 (16.7) | 3 (30.0) | 0 | 0 | 2 (100.0) | 0 | 1 (100) | 0 | 0 | 12 (22.2) |
| Doing schoolwork/ homework | 3 (15.8) | 1 (16.7) | 2 (20.0) | 0 | 0 | 1 (50.0) | 0 | 0 | 0 | 0 | 7 (13.0) |
| Spending time with my friends | 6 (31.6) | 0 | 2 (20.0) | 0 | 1 (50.0) | 1 (50.0) | 0 | 0 | 0 | 0 | 10 (18.5) |
| Spending time with family | 4 (21.1) | 0 | 4 (40.0) | 0 | 1 (50.0) | 2 (100) | 0 | 0 | 1 (16.7) | 0 | 12 (22.2) |
| Sleeping | 7 (36.8) | 3 (50.0) | 4 (40.0) | 0 | 1 (50.0) | 2 (100) | 0 | 1 (100.0) | 1 (16.7) | 1 (50.0) | 20 (37.0) |
| Other | 1 (5.3) | 0 | 0 | 0 | 0 | 0 | 0 | 0 | 0 | 0 | 1 (1.9) |
| None of the above | 8 (42.1) | 2 (33.3) | 3 (30.0) | 0 | 0 | 0 | 4 (80.0) | 0 | 4 (66.7) | 0 | 21 (38.9) |

All data presented in this table are self-reported by survey participants

*ARG* Argentina, *BRA* Brazil, *COL* Colombia, *CRO* Croatia, *DEN* Denmark, *GER* Germany, *HUN* Hungary, *IRL* Ireland, *POL* Poland, *POR* Portugal

^a^One caregiver from Brazil filled the survey for two children; the caregiver was not asked questions about impact on daily activities twice

**Supplementary Table 6** Self-reported impact on relationships of caregiving to pediatric patients with HAE by country

| **Impact, n (%)** | **ARG**  **n=19** | **BRA**  **n=6^a^** | **COL**  **n=10** | **CRO**  **n=1** | **DEN**  **n=2** | **GER**  **n=2** | **HUN**  **n=5** | **IRL**  **n=1** | **POL**  **n=6** | **POR**  **n=2** | **Total**  **N=54^a^** |
| --- | --- | --- | --- | --- | --- | --- | --- | --- | --- | --- | --- |
| Difficulty spending as much time as I would like with family | 8 (42.1) | 1 (16.7) | 3 (30.0) | 0 | 1 (50.0) | 2 (100.0) | 0 | 0 | 1 (16.7) | 0 | 16 (29.1) |
| Difficulty spending as much time as I would like with friends | 8 (42.1) | 1 (16.7) | 1 (10.0) | 0 | 1 (50.0) | 2 (100.0) | 0 | 0 | 0 | 0 | 13 (23.6) |
| Difficulty maintaining my relationship with my spouse/partner | 8 (42.1) | 2 (33.3) | 1 (10.0) | 0 | 0 | 1 (50.0) | 1 (20.0) | 1 (100.0) | 1 (16.7) | 0 | 15 (27.3) |
| I have separated or divorced | 5 (26.3) | 1 (16.7) | 0 | 0 | 0 | 0 | 0 | 0 | 0 | 0 | 6 (10.9) |
| Difficulty maintaining friendships | 6 (31.6) | 1 (16.7) | 0 | 0 | 0 | 0 | 0 | 1 (100.0) | 0 | 0 | 8 (14.5) |
| Reduced intimacy with my spouse/partner | 7 (36.8) | 1 (16.7) | 0 | 0 | 0 | 0 | 0 | 1 (100.0) | 0 | 0 | 9 (16.4) |
| Lack of understanding from my spouse/partner about the time needed to care for my child with HAE | 6 (31.6) | 0 | 0 | 0 | 0 | 0 | 0 | 0 | 1 (16.7) | 0 | 7 (12.7) |
| Lack of understanding from my family about the time needed to care for my child with HAE | 6 (31.6) | 0 | 1 (10.0) | 0 | 0 | 0 | 0 | 0 | 0 | 0 | 7 (12.7) |
| Lack of understanding from my friends about the time needed to care for my child with HAE | 5 (26.3) | 0 | 1 (10.0) | 0 | 0 | 0 | 0 | 0 | 1 (16.7) | 0 | 7 (12.7) |
| Lack of understanding from my boss and/or co-workers about the time needed to care for my child with HAE | 7 (36.8) | 0 | 0 | 1 (100.0) | 0 | 1 (50.0) | 0 | 0 | 0 | 0 | 9 (16.4) |
| Lack of understanding from my child’s teachers and/or school about the time needed to care for my child with HAE | 6 (31.6) | 2 (33.3) | 0 | 0 | 0 | 0 | 1 (20.0) | 0 | 2 (33.3) | 0 | 11 (20.0) |
| Other | 0 | 0 | 0 | 0 | 0 | 0 | 0 | 0 | 0 | 0 | 0 |
| None of the above | 9 (47.4) | 4 (66.7) | 7 (70.0) | 0 | 1 (50.0) | 0 | 3 (60.0) | 0 | 4 (66.7) | 2 (100%) | 30 (55.5) |

All data presented in this table are self-reported by survey participants

*ARG* Argentina, *BRA* Brazil, *COL* Colombia, *CRO* Croatia, *DEN* Denmark, *GER* Germany, *HUN* Hungary, *IRL* Ireland, *POL* Poland, *POR* Portugal

^a^One caregiver from Brazil filled the survey for two children; the caregiver was not asked questions about impacts on relationships twice

**Supplementary Table 7** Self-reported impact on emotional well-being of caregiving to pediatric patients with HAE by country

| **Emotional Impacts, n (%)** | **ARG**  **n=19** | **BRA**  **n=6^a^** | **COL**  **n=10** | **CRO**  **n=1** | **DEN**  **n=2** | **GER**  **n=2** | **HUN**  **n=5** | **IRL**  **n=1** | **POL**  **n=6** | **POR**  **n=2** | **Total**  **N=54^a^** |
| --- | --- | --- | --- | --- | --- | --- | --- | --- | --- | --- | --- |
| Worrying about the patient’s health | 18 (94.7) | 5 (83.3) | 9 (90.0) | 0 | 2 (100.0) | 2 (100.0) | 5 (100.0) | 1 (100.0) | 5 (83.3) | 2 (100.0) | 49 (90.7) |
| Worrying about the future | 18 (94.7) | 3 (50.0) | 5 (50.0) | 0 | 2 (100.0) | 0 | 4 (80.0) | 1 (100.0) | 4 (66.7) | 0 | 37 (68.5) |
| Worrying about finances | 10 (52.6) | 2 (33.3) | 2 (20.0) | 0 | 0 | 0 | 1 (20.0) | 1 (100.0) | 2 (33.3) | 0 | 18 (33.3) |
| Anxiety | 15 (78.9) | 5 (83.3) | 4 (40.0) | 0 | 0 | 1 (50.0) | 2 (40.0) | 1 (100.0) | 1 (16.7) | 0 | 29 (53.7) |
| Sadness | 13 (68.4) | 2 (33.3) | 7 (70.0) | 0 | 2 (100.0) | 2 (100.0) | 0 | 0 | 1 (16.7) | 0 | 27 (50.0) |
| Anger | 12 (63.2) | 2 (33.3) | 1 (10.0) | 0 | 1 (50.0) | 1 (50.0) | 0 | 0 | 0 | 0 | 17 (31.5) |
| Frustration | 9 (47.4) | 3 (50.0) | 4 (40.0) | 0 | 1 (50.0) | 1 (50.0) | 1 (20.0) | 0 | 0 | 0 | 19 (35.2) |
| Depression | 8 (42.1) | 1 (16.7) | 2 (20.0) | 0 | 0 | 0 | 0 | 0 | 0 | 0 | 11 (20.4) |
| Stress | 8 (42.1) | 3 (50.0) | 4 (40.0) | 1 (100.0) | 0 | 2 (100.0) | 4 (80.0) | 0 | 1 (16.7) | 0 | 23 (42.6) |
| Feeling helpless | 7 (36.8) | 4 (66.7) | 1 (10.0) | 0 | 1 (50.0) | 1 (50.0) | 2 (40.0) | 0 | 3 (50.0) | 0 | 19 (35.2) |
| Resentment | 6 (31.6) | 2 (33.3) | 0 | 0 | 0 | 1 (50.0) | 0 | 0 | 1 (16.7) | 0 | 10 (18.5) |
| Guilt | 10 (52.6) | 2 (33.3) | 0 | 0 | 2 (100.0) | 1 (50.0) | 3 (60.0) | 1 (100.0) | 0 | 0 | 19 (35.2) |
| None of the above | 1 (5.3) | 1 (16.7) | 0 | 0 | 0 | 0 | 0 | 0 | 0 | 0 | 2 (3.7) |

All data presented in this table are self-reported by survey participants

*ARG* Argentina, *BRA* Brazil, *COL* Colombia, *CRO* Croatia, *DEN* Denmark, *GER* Germany, *HUN* Hungary, *IRL* Ireland, *POL* Poland, *POR* Portugal

^a^One caregiver from Brazil filled the survey for two children; the caregiver was not asked questions about impact on emotional well-being twice

**Supplementary Table 8** Caregiver-reported clinical characteristics of adult patients with HAE by country

| Characteristic | ARG  n=29 | BRA  n=3 | COL  n=14 | DEN  n=7 | GER  n=3 | HUN  n=4 | IRL  n=2 | POR  n=3 | SWE  n=1 | Total  N=66 |
| --- | --- | --- | --- | --- | --- | --- | --- | --- | --- | --- |
| Type of HAE, n (%) |  |  |  |  |  |  |  |  |  |  |
| HAE-C1INH-Type1 | 26 (89.7) | 2 (66.7) | 6 (42.9) | 5 (71.4) | 2 (66.7) | 2 (50.0) | 1 (50.0) | 1 (33.3) | 1 (100.0) | 46 (69.7) |
| HAE-C1INH-Type2 | 0 | 0 | 4 (28.6) | 1 (14.3) | 0 | 1 (25.0) | 1 (50.0) | 0 | 0 | 7 (10.6) |
| HAE-C1INH undifferentiated^a^ | 2 (6.9) | 1 (33.3) | 2 (14.3) | 1 (14.3) | 1 (33.3) | 1 (25.0) | 0 | 2 (66.7) | 0 | 10 (15.2) |
| Unknown^b^ | 1 (3.4) | 0 | 2 (14.3) | 0 | 0 | 0 | 0 | 0 | 0 | 3 (4.5) |
| Current LTP use, n (%) |  |  |  |  |  |  |  |  |  |  |
| Yes | 15 (51.7) | 2 (66.7) | 9 (64.2) | 5 (71.4) | 1 (33.3) | 2 (50) | 2 (100.0) | 3 (100.0) | 1 (100.0) | 40 (60.6) |
| No | 14 (48.2) | 1 (3.3) | 4 (28.6) | 2 (28.6) | 2 (66.7) | 2 (50) | 0 | 0 | 0 | 25 (37.9) |
| Unknown | 0 | 0 | 1 (7.1) | 0 | 0 | 0 | 0 | 0 | 0 | 1 (1.5) |

All data presented in this table are self-reported by survey participants

*ARG* Argentina, *BRA* Brazil, *COL* Colombia, *DEN* Denmark, *GER* Germany, *HAE* hereditary angioedema, *HAE-C1INH* hereditary angioedema due to C1 inhibitor deficiency, *HUN* Hungary, *IRL* Ireland, *LTP* long-term prophylaxis, *POR* Portugal, *SWE* Sweden

^a^Includes patients for whom the caregiver selected the answer “Unsure of exact HAE type, but it is either HAE Type I or II” to the survey question “Which type of HAE does the patient have?”

^b^Includes patients for whom the caregiver selected the answer “I don’t know what type of HAE” to the survey question “Which type of HAE does the patient have?”

**Supplementary Table 9** Type of care typically provided by caregivers of adult patients with HAE

| **Types of help, n (%)** | **ARG**  **n=29** | **BRA**  **n=3** | **COL**  **n=14** | **DEN**  **n=7** | **GER**  **n=3** | **HUN**  **n=4** | **IRL**  **n=2** | **POR**  **n=3** | **SWE**  **n=1** | **Total**  **N=66** |
| --- | --- | --- | --- | --- | --- | --- | --- | --- | --- | --- |
| Help with household chores | 11 (37.9) | 3 (100.0) | 11 (78.6) | 3 (42.9) | 2 (66.7) | 2 (50.0) | 0 | 2 (66.7) | 0 | 34 (51.5) |
| Help with cooking | 11 (37.9) | 2 (66.7) | 5 (35.7) | 4 (57.1) | 2 (66.7) | 2 (50.0) | 0 | 2 (66.7) | 0 | 28 (42.4) |
| Help with grocery shopping | 12 (41.4) | 2 (66.7) | 6 (42.9) | 4 (57.1) | 2 (66.7) | 3 (75.0) | 0 | 2 (66.7) | 0 | 31 (47.0) |
| Help with childcare | 9 (31.0) | 1 (33.3) | 6 (42.9) | 1 (14.3) | 1 (33.3) | 1 (25.0) | 1 (50.0) | 0 | 0 | 20 (30.3) |
| Help with laundry | 8 (27.6) | 2 (66.7) | 7 (50.0) | 3 (42.9) | 2 (66.7) | 1 (25.0) | 1 (50.0) | 1 (33.3) | 0 | 25 (37.9) |
| Providing or helping during transportation to doctors appointments | 13 (44.8) | 2 (66.7) | 10 (71.4) | 1 (14.3) | 2 (66.7) | 2 (50.0) | 1 (50.0) | 1 (33.3) | 0 | 32 (48.5) |
| Providing or helping during transportation to work or school | 3 (10.3) | 2 (66.7) | 5 (35.7) | 0 | 1 (33.3) | 0 | 1 (50.0) | 1 (33.3) | 0 | 13 (19.7) |
| Help with dressing or undressing | 3 (10.3) | 0 | 1 (7.1) | 0 | 1 (33.3) | 1 (25.0) | 0 | 1 (33.3) | 0 | 7 (10.6) |
| Help with bathing or showering | 2 (6.9) | 0 | 1 (7.1) | 0 | 1 (33.3) | 1 (25.0) | 0 | 0 | 0 | 5 (7.6) |
| Help with eating or drinking | 2 (6.9) | 1 (33.3) | 2 (14.3) | 0 | 1 (33.3) | 0 | 0 | 0 | 0 | 6 (9.1) |
| Help with grooming (hair care, shaving) | 2 (6.9) | 0 | 2 (14.3) | 0 | 1 (33.3) | 0 | 0 | 1 (33.3) | 0 | 6 (9.1) |
| Help with going to the toilet | 4 (13.8) | 0 | 1 (7.1) | 0 | 1 (33.3) | 0 | 0 | 0 | 0 | 6 (9.1) |
| Help with moving around the house or property | 1 (3.4) | 0 | 1 (7.1) | 0 | 1 (33.3) | 0 | 0 | 0 | 0 | 3 (4.5) |
| Help with paying bills or with completing forms and other paperwork | 6 (20.7) | 1 (33.3) | 3 (21.4) | 1 (14.3) | 1 (33.3) | 1 (25.0) | 0 | 1 (33.3) | 0 | 14 (21.2) |
| Help HAE medication | 18 (62.1) | 1 (33.3) | 5 (35.7) | 4 (57.1) | 1 (33.3) | 1 (25.0) | 1 (50.0) | 2 (66.7) | 1 (100.0) | 34 (51.5) |
| Reminding patient to take medications | 13 (44.8) | 2 (66.7) | 3 (21.4) | 5 (71.4) | 2 (66.7) | 1 (25.0) | 2 (100.0) | 2 (66.7) | 0 | 30 (45.5) |
| Scheduling or managing doctors appointments | 11 (37.9) | 1 (33.3) | 5 (35.7) | 1 (14.3) | 1 (33.3) | 1 (25.0) | 2 (100.0) | 1 (33.3) | 0 | 23 (34.8) |
| Emotional support | 24 (82.8) | 3 (100.0) | 11 (78.6) | 7 (100) | 2 (66.7) | 3 (75.0) | 2 (100.0) | 2 (66.7) | 1 (100.0) | 55 (83.3) |
| Spending time with patient | 22 (75.9) | 3 (100.0) | 10 (71.4) | 4 (57.1) | 3 (100.0) | 3 (75.0) | 1 (50.0) | 2 (66.7) | 1 (100.0) | 49 (74.2) |
| Advocating for the patient’s care | 14 (48.3) | 2 (66.7) | 4 (28.6) | 3 (42.9) | 3 (100.0) | 0 | 1 (50.0) | 2 (66.7) | 0 | 29 (43.9) |
| Other | 1 (3.4) | 0 | 0 | 0 | 0 | 0 | 0 | 0 | 0 | 1 (1.5) |

All data presented in this table are self-reported by survey participants

*ARG* Argentina, *BRA* Brazil, *COL* Colombia, *DEN* Denmark, *GER* Germany, *HUN* Hungary, *IRL* Ireland, *POR* Portugal, *SWE* Sweden

**Supplementary Table 10** Self-reported impact on daily activities of caregiving to adult patients with HAE by country

| Activities Impacted, n (%) | ARG  n=29 | BRA  n=3 | COL  n=14 | DEN  n=7 | GER  n=3 | HUN  n=4 | IRL  n=2 | POR  n=3 | SWE  n=1 | Total  N=66 |
| --- | --- | --- | --- | --- | --- | --- | --- | --- | --- | --- |
| Self-care | 1 (3.4) | 1 (33.3) | 1 (7.1) | 0 | 0 | 0 | 0 | 1 (33.3) | 0 | 4 (6.1) |
| Recreational activities | 9 (31.0) | 2 (66.7) | 3 (21.4) | 0 | 1 (33.3) | 1 (25.0) | 0 | 2 (66.7) | 0 | 18 (27.3) |
| Household chores | 5 (17.2) | 1 (33.3) | 3 (21.4) | 0 | 1 (33.3) | 1 (25.0) | 0 | 2 (66.7) | 0 | 13 (19.7) |
| Work responsibilities | 14 (48.3) | 0 | 2 (14.3) | 0 | 0 | 1 (25.0) | 0 | 2 (66.7) | 0 | 19 (28.8) |
| Running errands | 5 (17.2) | 2 (66.7) | 2 (14.3) | 0 | 1 (33.3) | 0 | 0 | 1 (33.3) | 0 | 11 (16.7) |
| Doing schoolwork/ homework | 1 (3.4) | 0 | 1 (7.1) | 0 | 0 | 0 | 0 | 2 (66.7) | 0 | 4 (6.1) |
| Spending time with my friends | 6 (20.7) | 2 (66.7) | 1 (7.1) | 0 | 0 | 1 (25.0) | 0 | 1 (33.3) | 0 | 11 (16.7) |
| Spending time with family | 5 (17.2) | 0 | 1 (7.1) | 0 | 0 | 1 (25.0) | 0 | 1 (33.3) | 0 | 8 (12.1) |
| Sleeping | 10 (34.5) | 1 (33.3) | 4 (28.6) | 0 | 0 | 2 (50.0) | 0 | 2 (66.7) | 0 | 19 (28.8) |
| Other | 0 | 0 | 0 | 0 | 0 | 0 | 0 | 0 | 0 | 0 |
| None of the above | 10 (34.5) | 1 (33.3) | 7 (50.0) | 7 (100.0) | 2 (66.7) | 2 (50.0) | 2 (100.0) | 1 (33.3) | 1 (100.0) | 33 (50.0) |

All data presented in this table are self-reported by survey participants

*ARG* Argentina, *BRA* Brazil, *COL* Colombia, *DEN* Denmark, *GER* Germany, *HUN* Hungary, *IRL* Ireland, *POR* Portugal, *SWE* Sweden

**Supplementary Table 11** Self-reported impact on future planning of caregiving to adult patients with HAE by country

| Plans/abilities impacted, n (%) | ARG  n=29 | BRA  n=3 | COL  n=14 | DEN  n=7 | GER  n=3 | HUN  n=4 | IRL  n=2 | POR  n=3 | SWE  n=1 | Total  N=66 |
| --- | --- | --- | --- | --- | --- | --- | --- | --- | --- | --- |
| Work full-time | 5 (17.2) | 0 | 2 (14.3) | 0 | 0 | 0 | 1 (50.0) | 0 | 0 | 8 (12.1) |
| Interests/hobbies | 5 (17.2) | 2 (66.7) | 1 (7.1) | 0 | 1 (33.3) | 0 | 1 (50.0) | 1 (33.3) | 0 | 11 (16.7) |
| Travel/vacations/ holidays | 5 (17.2) | 1 (33.3) | 1 (7.1) | 0 | 1 (33.3) | 2 (50.0) | 0 | 1 (33.3) | 1 (100.0) | 12 (18.2) |
| Move/relocate cities or countries | 3 (10.3) | 1 (33.3) | 0 | 0 | 1 (33.3) | 0 | 0 | 1 (33.3) | 1 (100.0) | 7 (10.6) |
| Have children | 0 | 1 (33.3) | 1 (7.1) | 0 | 2 (66.7) | 1 (25.0) | 0 | 0 | 0 | 5 (7.6) |
| Other | 0 | 0 | 0 | 0 | 0 | 0 | 0 | 0 | 0 | 0 |
| None of the above | 17 (58.6) | 0 | 10 (71.4) | 7 (100.0) | 1 (33.3) | 2 (50.0) | 1 (50.0) | 2 (66.7) | 0 | 40 (60.6) |

All data presented in this table are self-reported by survey participants

*ARG* Argentina, *BRA* Brazil, *COL* Colombia, *DEN* Denmark, *GER* Germany, *HUN* Hungary, *IRL* Ireland, *POR* Portugal, *SWE* Sweden

**Supplementary Table 12** Self-reported impact on emotional well-being of caregiving to adult patients with HAE by country

| Emotional Impacts, n (%) | ARG  n=29 | BRA  n=3 | COL  n=14 | DEN  n=7 | GER  n=3 | HUN  n=4 | IRL  n=2 | POR  n=3 | SWE  n=1 | Total  n=66 |
| --- | --- | --- | --- | --- | --- | --- | --- | --- | --- | --- |
| Worrying about the patient‘s health | 28 (96.6) | 3 (100.0) | 14 (100.0) | 5 (71.4) | 2 (66.7) | 4 (100.0) | 1 (50.0) | 3 (100.0) | 1 (100.0) | 61 (92.4) |
| Worrying about the future | 14 (48.3) | 1 (33.3) | 6 (42.9) | 1 (14.3) | 1 (33.3) | 2 (50.0) | 0 | 1 (33.3) | 1 (100.0) | 27 (40.9) |
| Worrying about finances | 3 (10.3) | 2 (66.7) | 2 (14.3) | 0 | 0 | 1 (25.0) | 1 (50.0) | 2 (66.7) | 0 | 11 (16.7) |
| Anxiety | 6 (20.7) | 0 | 4 (28.6) | 0 | 2 (66.7) | 2 (50.0) | 1 (50.0) | 2 (66.7) | 0 | 17 (25.8) |
| Sadness | 7 (24.1) | 1 (33.3) | 4 (28.6) | 1 (14.3) | 1 (33.3) | 1 (25.0) | 0 | 1 (33.3) | 1 (100.0) | 17 (25.8) |
| Anger | 2 (6.9) | 1 (33.3) | 0 | 0 | 0 | 1 (25.0) | 0 | 1 (33.3) | 0 | 5 (7.6) |
| Frustration | 5 (17.2) | 1 (33.3) | 5 (35.7) | 1 (14.3) | 0 | 2 (50.0) | 0 | 2 (66.7) | 1 (100.0) | 17 (25.8) |
| Depression | 1 (3.4) | 0 | 2 (14.3) | 0 | 0 | 0 | 1 (50.0) | 1 (33.3) | 0 | 5 (7.6) |
| Stress | 8 (27.6) | 1 (33.3) | 3 (21.4) | 0 | 2 (66.7) | 2 (50.0) | 0 | 2 (66.7) | 0 | 18 (27.3) |
| Feeling helpless | 3 (10.3) | 1 (33.3) | 0 | 2 (28.6) | 1 (33.3) | 2 (50.0) | 1 (50.0) | 2 (66.7) | 1 (100.0) | 13 (19.7) |
| Resentment | 0 | 0 | 0 | 0 | 0 | 1 (25.0) | 0 | 1 (33.3) | 0 | 2 (3.0) |
| Guilt | 4 (13.8) | 0 | 3 (21.4) | 0 | 0 | 2 (50.0) | 1 (50.0) | 0 | 0 | 10 (15.2) |
| Other | 0 | 0 | 0 | 0 | 0 | 0 | 0 | 0 | 0 | 0 |
| None of the above | 1 (3.4) | 0 | 0 | 1 (14.3) | 1 (33.3) | 0 | 1 (50.0) | 0 | 0 | 4 (6.1) |

All data presented in this table are self-reported by survey participants

*ARG* Argentina, *BRA* Brazil, *COL* Colombia, *DEN* Denmark, *GER* Germany, *HUN* Hungary, *IRL* Ireland, *POR* Portugal, *SWE* Sweden
